# Exploiting the neoantigen landscape for immunotherapy of pancreatic ductal adenocarcinoma

**DOI:** 10.1038/srep35848

**Published:** 2016-10-20

**Authors:** Peter Bailey, David K. Chang, Marie-Andrée Forget, Francis A. San Lucas, Hector A. Alvarez, Cara Haymaker, Chandrani Chattopadhyay, Sun-Hee Kim, Suhendan Ekmekcioglu, Elizabeth A. Grimm, Andrew V. Biankin, Patrick Hwu, Anirban Maitra, Jason Roszik

**Affiliations:** 1 Wolfson Wohl Cancer Research Centre, Institute for Cancer Sciences, University of Glasgow, Garscube Estate, Bearsden, Glasgow G61 1BD, UK; 2 West of Scotland Pancreatic Unit, Glasgow Royal Infirmary, Glasgow G31 2ER, United Kingdom; 3 Department of Surgery, Bankstown Hospital, Eldridge Road, Bankstown, Sydney, New South Wales 2200, Australia; 4 South Western Sydney Clinical School, Faculty of Medicine, University of New South Wales, Liverpool, New South Wales 2170, Australia; 5 Department of Melanoma Medical Oncology, The University of Texas MD Anderson Cancer Center, 1515 Holcombe Blvd., Houston, TX, 77030, USA; 6 Departments of Pathology and Translational Molecular Pathology, Ahmed Center for Pancreatic Cancer Research, The University of Texas MD Anderson Cancer Center, 1515 Holcombe Blvd., Houston, TX, 77030, USA; 7 Department of Genomic Medicine, The University of Texas MD Anderson Cancer Center, 1515 Holcombe Blvd., Houston, TX, 77030, USA

## Abstract

Immunotherapy approaches for pancreatic ductal adenocarcinoma (PDAC) have met with limited success. It has been postulated that a low mutation load may lead to a paucity of T cells within the tumor microenvironment (TME). However, it is also possible that while neoantigens are present, an effective immune response cannot be generated due to an immune suppressive TME. To discern whether targetable neoantigens exist in PDAC, we performed a comprehensive study using genomic profiles of 221 PDAC cases extracted from public databases. Our findings reveal that: (a) nearly all PDAC samples harbor potentially targetable neoantigens; (b) T cells are present but generally show a reduced activation signature; and (c) markers of efficient antigen presentation are associated with a reduced signature of markers characterizing cytotoxic T cells. These findings suggest that despite the presence of tumor specific neoepitopes, T cell activation is actively suppressed in PDAC. Further, we identify iNOS as a potential mediator of immune suppression that might be actionable using pharmacological avenues.

Pancreatic ductal adenocarcinoma (PDAC) will be diagnosed in approximately 53,000 patients in the United States in 2016, and an estimated 41,000 will die from its effects^1^. One the most lethal forms of cancer, the overall five year survival rate of patients with PDAC is only 8%. A large proportion of patients present with advanced and metastatic disease at initial diagnosis and are presented with treatment options restricted to cytotoxic chemotherapies which extend life by no more than a few months^2^. A significant amount of research aims to identify new therapeutic agents and effective combinations of existing therapies to generate more options for PDAC patients at all stages of disease, but these efforts have not yet significantly improved patient survival^3^.

Immunotherapy has proven to be a promising therapeutic avenue for numerous solid cancers, such as melanoma and lung cancers. Cancers express tumor-associated antigens (TAAs), short peptides of 8–12 amino acids, on human leukocyte antigen (HLA) molecules at the cell surface. TAAs can be neoantigens, arising as a result of processed cancer-specific mutant peptides, or aberrant self-antigens like mesothelin or epidermal growth factor receptor (EGFR), due to overexpression of oncogenic proteins. Neoantigens usually evoke a more robust T-cell response due to the lack of thymic elimination of autoreactive T cells, which is known to exist against self-antigens. Not surprisingly, cancers with higher mutation loads also harbor higher numbers of neontigens presented on HLA molecules, thereby leading to an influx of greater numbers of TAA-reactive T cells. For example, melanomas demonstrate an average of 511 coding mutations according to Cancer Genome Atlas (TCGA) data, and are characterized by large numbers of tumor-infiltrating lymphocytes (TILs)^4^. Another example of this correlation between mutational load and TILs is observed in cancers with microsatellite instability (e.g., Lynch syndrome), where large numbers of TILs are observed in cases that bear thousands of neoantigens^5^.

Clinical trials of immune checkpoint inhibitors (anti-CTLA-4) and cancer vaccines (e.g. MUC1, GVAX), although well tolerated and able to generate an immune response, have demonstrated only limited impact on overall patient survival in PDAC^6^,^7^,^8^,^9^,^10^,^11^. Data from animal models of PDAC suggest a phenomenon of T-cell exclusion from the immediate tumor microenvironment (TME), which might be the basis for recalcitrance to checkpoint inhibitor therapy (including, anti-PD-1 and anti-CTLA-4)^12^. A possible reason for this T-cell exclusion may be the relatively low mutational load observed in PDAC (we identified an average of 62 coding mutations in our recent study on genomic analysis of PDAC^13^). An alternative explanation for the resistance of PDAC to immunotherapy may be that while both neoantigens and corresponding TAA-reactive CD8^+^ TILs exist in PDAC, there is profound immune suppression that leads to ineffective T-cell activation and immune rejection. Such functional immune suppression, despite an effector T-cell influx, might be caused by the presence of regulatory T cells (Tregs), myeloid-derived suppressor cells (MDSCs), tumor-associated macrophages (TAMs) and other inhibitory cytokines like TGFbeta and IL-10, all of which have been reported in the PDAC milieu^14^,^15^. In such cases, removal of immune suppression barriers, such as depletion of myeloid cells^16^, or artificially “boosting” the immune response through TAA-targeted vaccines or adoptively transferring T cells could enable overcoming tolerance^17^,^18^. In line with this, it has recently been shown that T cells engineered against Mesothelin, a surface antigen present on mesothelial cells, can cause tumor lysis and increased survival in PDAC patients^19^.

The objective of this study was to use large publicly available datasets of PDAC – specifically, TCGA and the Australian Pancreatic Cancer Genome Initiative, Australian contribution to the International Cancer Genome Consortium (ICGC) – to comprehensively decipher the neoantigen landscape of PDAC, and to determine how this landscape correlates with the measurable immune response signatures within the TME. For the first time, availability of deeply sequenced transcriptomes derived from “whole tissue” human PDAC samples (*versus* cell line or xenograft-derived RNA profiles) allows us to address questions related to both cancer cell specific neoantigens, as well as the host immune response, in this disease. Auditing the neoantigen landscape of PDAC is significant, given the possibility of identifying recurrent epitopes that might be presented across multiple tumors that share HLA subtypes. This finding, in turn, could enable the development of shared TAA-targeted vaccines or adoptive cellular approaches and to augment the possibility of immune rejection, as previously mentioned. An additional deliverable of our approach is to identify putative mediators of immune suppression within the TME, which might be impeding an effective T-cell response in tumors with an otherwise discernible TIL influx. Indeed, as further elaborated below, we find that PDAC cases, while lacking the neoantigen diversity of melanomas, do present candidate neoantigens, including ones expected to have efficient presentation by HLA class I molecules. Further, in contrast to some prior reports in animal models, we find robust TIL signatures within the TME, but a reduced expression of activation associated transcripts, suggesting a functional immune suppression as the dominant outcome. Finally, we identify some potential mediators of this immune suppression that might be targetable using pharmacological agents, possibly in combination with immune therapies to overcome the profound recalcitrance to immune response in PDAC.

## Results

### Nearly all PDAC cases express candidate neoantigens

**Figure 1:**
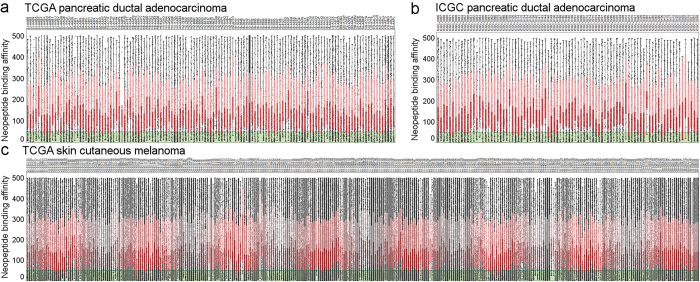
The neoantigen landscape of pancreatic ductal adenocarcinoma and skin cutaneous melanoma. Binding affinity of neopeptides is shown for PDAC samples in the TCGA (**a**) and ICGC (**b**) projects for affinity <500 nM neopeptides. Strong binder (<50 nM) neopeptides are highlighted with green color. TCGA skin cutaneous melanoma neopeptides are presented similary (**c**). Red boxes represent the quartile below the median neopeptide binding affinity, while pink boxes highlight quartiles above the median binding affinity.

A database was constructed of all possible neopeptides that are predicted to bind to, and be presented by, a given patient’s HLA molecules. The landscape of neoantigens (Fig. 1) in PDAC is not as vast as in skin cutaneous melanoma (SKCM): the TCGA (n = 133) and also the ICGC (n = 88)^20^ PDAC cases have significantly fewer predicted neoantigens than the TCGA SKCM tumors (n = 344). The predicted number of neoantigens varied from four to four thousand in PDAC samples. In SKCM the predicted potential neoantigen count was from eleven (which is considered very low in SKCM) to 14 thousand, and this was significantly higher than in PDAC (p < 0.001 for both PDAC data sets vs. SKCM). Notably, *KRAS* codon 12 mutations are the most common genetic alteration in this cancer, and indeed, the corresponding mutant peptide was predicted to generate likely immunogenic^21^ neoantigens for most of the TCGA and ICGC patients bearing this mutation (Supplementary Fig. 1). Thus, this mutant epitope could potentially be used as one target of an “off-the-shelf” vaccine or adoptively transferred T cells, although we do not have functional data on the avidity of the corresponding T-cell receptor (TCR). Other examples of mutations that we found to generate potential neoantigens for multiple patients were KRAS Q61H, TP53 R273N, TP53 R282W, MT-ND4 A318T, FCGBP A2493V, RBM12 P693S, and RET A1019V. Although the frequency of shared neoantigens from these mutations is low, these targets may be appropriate for a few percent of patients (Supplementary Fig. 2).

### PDAC is characterized by robust TILs, but absence of T-cell activation signature

**Figure 2:**
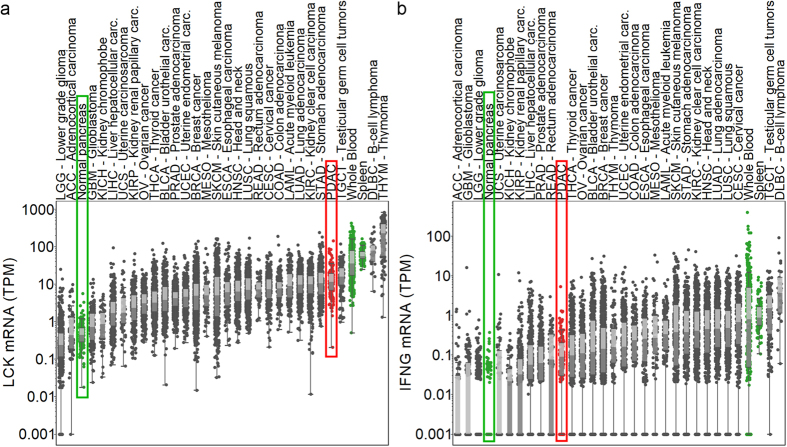
T cells are present but inactive in PDAC. Expression levels of LCK T-cell marker (**a**) and interferon gamma which plays a central role in immune system function (**b**) are shown on the y axis for 30 tumor tissue types (grey, red: pancreatic cancer) and normal tissues (normal pancreas, spleen, whole blood). Dark and light grey boxes represent the two quartiles around the median expression.

We analyzed the expression of LCK, a T-cell marker, and several genes related to T-cell function and subset (CD8A, IFNG, PRF1, GZMA, GZMB, CTLA4, PD-1, PD-L1, IDO1, IDO2, ARG1, LAG3, CD160, KLRG1) as well as Treg (FOXP3, CD25/IL2RA), and MDSC (CD11b, CD33, CD14, CD15) markers in TCGA tumor and Genotype-Tissue Expression (GTEx)^22^ normal tissues. We found very high PDAC LCK expression (Fig. 2a) indicating a robust TILs presence in PDAC. LCK expression in PDAC was not statistically significantly different from SKCM in the TCGA (Fig. 2a). In contrast, interferon gamma (IFNγ) expression was low (Fig. 2b) suggesting reduced T-cell activity. Effector T-cell markers and genes related to checkpoint blockade also showed lower expression in PDAC than in more immunogenic tumors like skin cutaneous melanoma, and higher expression than in normal pancreas (Supplementary Fig. 3, sample level figures and heatmap) further reinforcing the presence of less-activated T cells. CD8A, PRF1, GZMB, CTLA4, PD-1, LAG3, and CD160 expression was significantly lower in PDAC than in SKCM (p < 0.001 for each) similarly to the KLRG1 T-cell senescence marker (p < 0.05), but interestingly GZMA, IDO2, and ARG1 were significantly higher in PDAC than in SKCM (p < 0.001, p < 0.5, p < 0.001, respectively). The Treg transcription factor FOXP3 was significantly lower (p < 0.01), while CD25 (IL2RA) was significantly higher (p < 0.0001) in PDAC (n = 147) than in skin cutaneous melanoma (n = 472) indicating that CD25 is expressed in PDAC by non-regulatory T cells as well (Supplementary Fig. 3). HLA-A and the HLA expression regulator NLRC5^23^ showed high expression levels (Supplementary Fig. 3) further demonstrating that antigens can be presented in PDAC.

### Negative correlation between antigen presentation and immune cytotoxic activity

**Figure 3:**
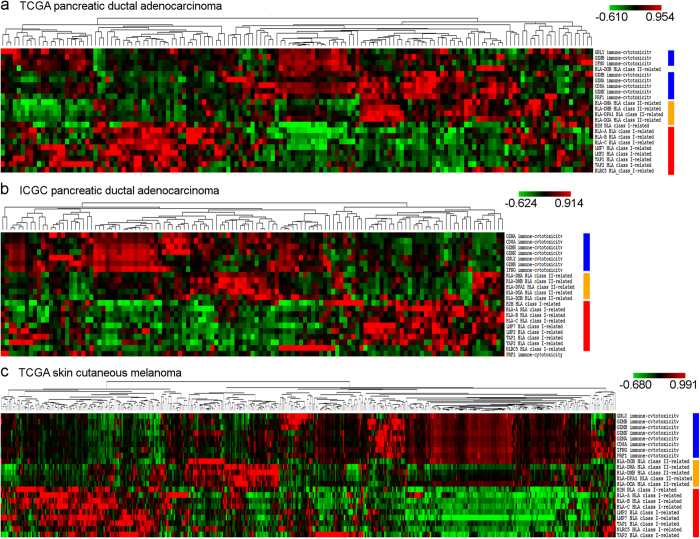
Gene expressions related to antigen presentation and cytotoxic activity are negatively correlated. Antigen presentation (red bar: class I-related - HLA-A, HLA-B, HLA-C, B2M, TAP1, TAP2, NLRC5, LMP2, and LMP7, orange bar: class II-related - HLA-DMA, HLA-DMB, HLA-DOA, HLA-DOB, and HLA-DPA1) and immune cytotoxicity gene expression (blue bar, CD8A, GNLY, GZMA, GZMB, GZMH, GZMK, IFNG, and PRF1) clustering shows two dominant cluster pairs in the TCGA (**a**) and ICGC (**b**) PDAC data sets, and also in SKCM (**c**). Low class I and/or class II antigen presentation (green in heat map) is associated with high (red in heat map) expression of cytotoxicity markers, while high antigen presentation correlates with low cytotoxic activity.

Clustering of expressions of cytotoxicity immune markers^24^, as well as antigen presentation-related genes showed a negative correlation between antigen presentation and immune activity (Fig. 3). High expression of HLA class I and class II genes were associated with low expression of cytotoxicity markers (CD8A, IFNG, PRF1, GZMA, GZMB) while low antigen presentation was coupled with higher immune activity in both the TCGA (Fig. 3a) and ICGC PDAC data sets (Fig. 3b). We also observed similar tendencies in the SKCM data set (Fig. 3c).

### High mutation load is associated with fewer cytotoxic T cells

**Figure 4:**
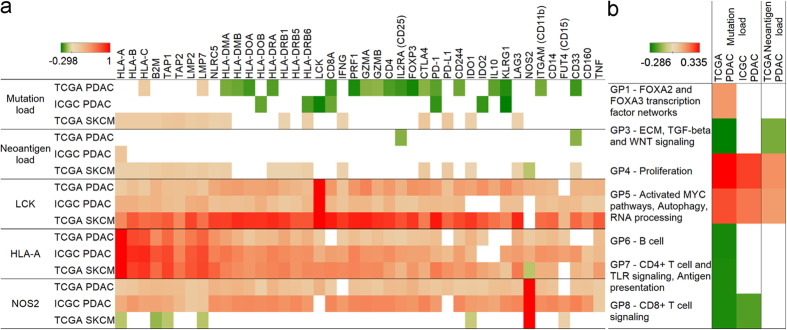
Mutation load in PDAC negatively correlates with T-cell activity. Spearman’s rank correlation coefficients showing how mutation load (first row), neoantigen load (second row), and LCK, HLA-A, and NOS2 (rows 3–5) correlate with gene expressions related to class I and II antigen presentation, cytotoxic activity, immune checkpoints, Treg, and MDSC cells (panel a). Gene program scores related to proliferation, activated MYC pathways, autophagy, and RNA processing show positive, while B cell, CD4+ T cell and TLR signaling, antigen presentation, and CD8+ T cell signaling show negative correlations (panel b). Only p < 0.1 correlations are shown.

Similarly to how antigen presentation-related gene expressions inversely correlated with immune cytotoxicity, mutation load also showed a negative correlation with cytotoxic immune markers (Fig. 4a). Notably, CD8A expression exhibited a negative correlation with mutation load in the TCGA (p < 0.01) and in the ICGC (p = 0.0817) PDAC data sets. Most of the class II (but not class I) antigen presentation and effector T-cell markers correlated negatively with mutation load in the TCGA pancreatic data set, and a few of them in the ICGC cohort as well. In line with this, we observed a significantly better (p < 0.05) overall survival for TCGA pancreatic patients having fewer mutations (Supplementary Fig. 4). Contrary to PDAC, skin cutaneous melanoma mutation and neoantigen load showed positive correlations with class I antigen presentation (Fig. 4a). LCK and HLA-A displayed similarly positive correlations in both cancers with all markers except NOS2 (iNOS), which was observed only in the two PDAC data sets. Clearly, NOS2 mRNA showed a positive correlation with antigen presentation and immune markers only in PDAC (Fig. 4a, last row) supporting that it plays a role in PDAC immunosuppression as the T-cell numbers increase in the tumor.

We have also correlated gene program (GP) scores^13^, which represent distinct biological processes, with mutation and neoantigen load. GP6, GP7 and GP8 scores (which represent immune programs) negatively correlated with mutation load (Fig. 4b). Interestingly, we found positive correlations between mutation and neoantigen load with GP4 (proliferation) and GP5 (activated MYC pathways, autophagy, and RNA processing) programs.

### NOS2 (iNOS) is overexpressed in PDAC and is a potential target to augment immune response

**Figure 5:**
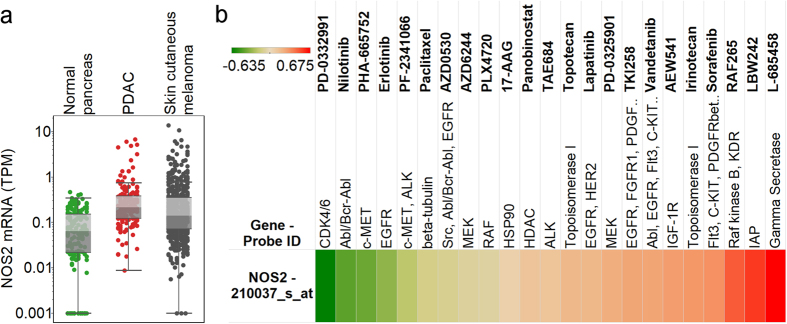
Expression and drug response associations of NOS2/iNOS. Gene expressions are shown on the y axis for normal pancreas, PDAC, and skin cutaneous melanoma (**a**). PDAC expression is significantly higher compared to normal pancreas (p < 0.0001). Pearson’s correlation coefficients of NOS2 gene expression and drug EC50 values (**b**) are presented as indicated by the color scale (green: negative, red: positive correlation).

NOS2 was the only immune suppression indicator which showed PDAC-specific correlations with immune markers, therefore we further characterized its role in PDAC. NOS2 expression in PDAC (n = 147) was significantly higher than in normal pancreas (n = 171) (p < 0.0001), while the pancreatic ductal adenocarcinoma and skin cutaneous melanoma (n = 472) expressions were not significantly different (Fig. 5a). NOS2 was expressed in the Cancer Cell Line Encyclopedia (CCLE) pancreatic cancer cell lines (Supplementary Fig. 5), therefore it is not only present in stromal and immune cells but also in the tumor cells. To show that NOS2 is functionally important in PDAC tumor cells we evaluated the drug response data of the CCLE and found that multiple drugs are more or less effective depending on the expression of NOS2 (Fig. 5b). Notably, CDK4/6 and c-MET inhibitors showed negative correlations between NOS2 expression and drug EC50 values.

## Discussion

We have conducted a systematic study to identify novel target antigens for PDAC immunotherapy. In the process, we have expanded and refined the repertoire of potential targetable PDAC neoantigens, identifying mutated peptides that could be used to maximize destruction of tumors with minimal to no damage to healthy tissues. We believe that a significant percentage of pancreatic cancer patients may benefit from immunotherapy approaches tailored to and targeting these PDAC-specific antigens. The most promising neoantigen candidate is from KRAS codon 12 mutations. It was recently shown that T-cell receptors (TCRs) that are reactive to KRAS G12V and G12D neoantigens can be isolated^21^.

Our results indicate that T cell-based immunotherapy approaches could be feasible for most patients with a KRAS G12 mutation. Other mutations may also give rise to similarly immunogenic mutations, but these would only be suitable for a few percent of patients. Our goal was to characterize the neoantigen universe of PDAC, however, it also needs to be determined which neopeptides are actually presented on HLA molecules of which patients. To do this, tumor-associated peptides can be eluted from MHC class I HLA-A, HLA-B, and HLA-C molecules isolated from PDAC tumor samples^25^,^26^.

In addition to demonstrating the availability of neoantigens, we have also shown that higher mutation load and a higher expression of genes involved in antigen presentation are associated with an expression profile that implies less cytotoxic activity. This is surprising because high HLA and neoantigen expression is associated with better prognosis in other cancers^27^. Although T cells are present in PDAC, they do not show a sufficient activity presumably due to immune suppression^28^. This mechanism also potentially includes immune-escape mechanisms such as decreasing the efficiency of neoantigen presentation in tumors where the cytotoxic T-cell infiltration is higher. Nevertheless, our results suggest that overexpression of the immunosuppressive inducible NO synthase (iNOS/NOS2) is associated with higher levels of antigen presentation and poor cytotoxic T-cell capability.

Many human cancers express inflammatory molecules that lead to an intrinsic pro-oxidant environment in the cancer cell, as well as potentiating a microenvironment that drives immune escape and resistance to apoptosis, one of which is iNOS. The concentration of NO, produced by iNOS, influences the antitumor immune response with increased NO associated immune suppression. Immune cells are functionally affected including the recruitment and/or activation of MDSCs, Tregs, tumor-associated macrophages, and Th2 lymphocytes. Once activated, MDSCs also produce high amounts of NO in the tumor microenvironment, which further contributes to the inhibition of antitumor responses and nitrosative stress^29^. It was also shown that targeting NO production may reverse MDSC-mediated immunosuppression by blocking inflammation-driven MDSC recruitment to the tumor^30^. The expression of iNOS in tumor cells associates with poor survival in several cancers^31^,^32^,^33^ and it is a known marker of chronic inflammation. Furthermore, fibroblasts express a significant amount of iNOS and this contributes to development of chemoresistance in pancreatic cancer cells through increased secretion of NO, which also leads to the release of IL-1beta by the tumor cells^34^. The immunosuppressive role of IL-1beta has already been shown in melanoma^35^. Our results indicate that higher iNOS expression is associated with poor T-cell effector function potentially limiting the effect of T cell-based immunotherapies. Multiple iNOS inhibitors have been developed^36^, and using these for the treatment of pancreatic cancer may help to prevent immune suppression and support adoptive T-cell therapies and cancer vaccines targeting the neoantigen landscape. Furthermore, iNOS inhibitors may also serve as effective combination partners in checkpoint blockade-based therapies. As Flavopiridol, a pan-CDK inhibitor, has been shown to downregulate iNOS expression^37^, and c-Met signaling possibly acts as a regulator of the NF-κB-iNOS-NO pathway^38^, we believe that the negative correlations between iNOS expression and CDK4/6 and c-MET inhibitor EC50 values indicate that iNOS plays an important role in PDAC tumor cells.

In summary, we have shown that nearly all PDAC tumors express potential targetable neoantigens which leads to a T-cell influx similar to immunogenic cancers like melanoma, however, an effective immune response cannot be generated due to a profoundly immune suppressive TME and potential immune escape. Unexpectedly, we found that markers of efficient antigen presentation are associated with a reduced signature of cytotoxic T cells, further suggesting an immune suppression mechanism that correlates with tumor antigenicity. Lastly, we identified high levels of immune-suppressive iNOS (NOS2) as a potential mediator of immune suppression, and targeting iNOS could help to augment immune response in PDAC.

## Methods

### Data sets

Gene expression, mutation, and clinical data from the pancreatic cancer TCGA were obtained from public TCGA repositories (https://tcga-data.nci.nih.gov and http://gdac.broadinstitute.org/). Raw sequencing data were requested though dbGaP (www.ncbi.nlm.nih.gov/gap) and from the authors of ICGC publications^13^,^20^. To analyze normal tissue expressions we used mRNA expression data from RNA sequencing from the Genotype-Tissue Expression (GTEx, http://www.gtexportal.org)^22^ project. CCLE cell line mRNA expression data were obtained from the Cancer Cell Line Encyclopedia^39^.

### Neoantigen predictions

We predicted all possible 8–12-mer mutated neo-peptides and their respective HLA-binding affinities (HLA-A, -B, -C) as described earlier^40^. We used mutation data from pancreatic ductal adenocarcinoma samples of the TCGA and ICGC^20^ projects. We applied the NetMHCpan (version 2.8)^41^ algorithm to predict peptide binding affinities to HLA-A, -B, and -C alleles. Patient HLA genotypes were determined from raw exome (normal and also tumor) and RNA sequencing data. We used the following HLA prediction tools: the Athlates software (version 2014_04_26)^42^ and the seq2HLA (version 2.2)^43^ program. We considered a neopeptide strong binder if binding affinity was < 50 nM, and weak binder if binding affinity was < 500 nM. We included both strong and weak binder neoantigens in our correlation analyses.

### RNA sequencing data analysis

Analysis of raw RNA sequencing data was performed using the RSEM (version 1.2.22) software^44^. We used the transcripts per million (TPM) unit, which was found the most appropriate to compare expression results from RNA sequencing^45^, to analyze ICGC, TCGA, and GTEx data. Gene program scores were generated as previously described^13^.

### Clustering and heatmap generation

Clustering of mRNA expression data and heatmap generation were performed using the Cluster and Treeview programs^46^.

### Other computational analyses

Pearson’s and Spearman’s rank correlation coefficients were calculated using the R software. Kaplan-Meier analyses were performed using the ‘survival’ R package. Comparisons of two groups used two-tailed Student’s t-tests assuming unequal variance. Differences were considered significant when p < 0.05, and a trend towards significance was shown when p < 0.1.

## Additional Information

**How to cite this article**: Bailey, P. *et al*. Exploiting the neoantigen landscape for immunotherapy of pancreatic ductal adenocarcinoma. *Sci. Rep.* **6**, 35848; doi: 10.1038/srep35848 (2016).

## Supplementary Material

Supplementary Information
