# Supplementary material for: Exploiting the neoantigen landscape for immunotherapy of pancreatic ductal adenocarcinoma
Source: Sci Rep. 2016 Oct 20;6:35848. doi: 10.1038/srep35848 (PMC5071896; doi:10.1038/srep35848)
Supplement: Supplementary Information [file srep35848-s1.pdf]

Exploiting the neoantigen landscape for immunotherapy of pancreatic ductal adenocarcinoma

Peter Bailey, David K. Chang, Marie-Andrée Forget, Francis A. San Lucas, Hector A. Alvarez, Cara Haymaker, Chandrani Chattopadhyay, Sun-Hee Kim, Suhendan Ekmekcioglu, Elizabeth A. Grimm, Andrew V. Biankin, Patrick Hwu, Anirban Maitra, Jason Roszik

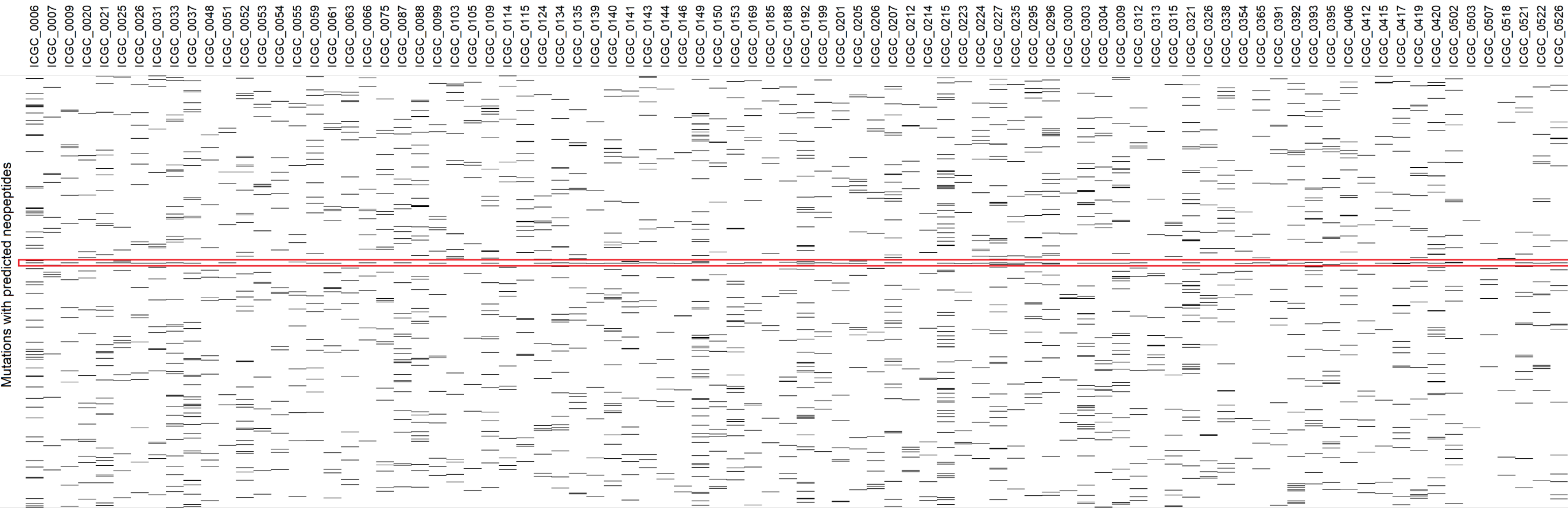

Supplementary Figure 1: PDAC mutations giving rise to neoantigens. The x axis shows the ICGC PDAC patients for which we predicted all neopeptides. The y axis represents the gene-mutation pairs which generate neopeptides predicted to have < 500 nM binding affinity. KRAS G12 mutations are highlighted in the red rectangle.

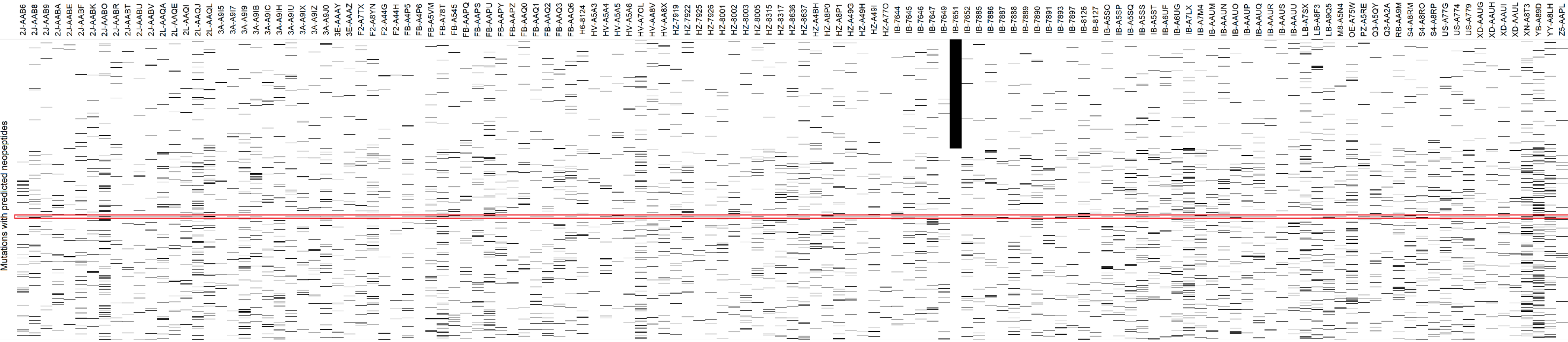

Supplementary Figure 1 (cont.): PDAC mutations giving rise to neoantigens. The x axis shows the TCGA PDAC patients for which we predicted all neopeptides. The y axis represents the gene-mutation pairs which generate neopeptides predicted to have < 500 nM binding affinity. KRAS G12 mutations are highlighted in the red rectangle.

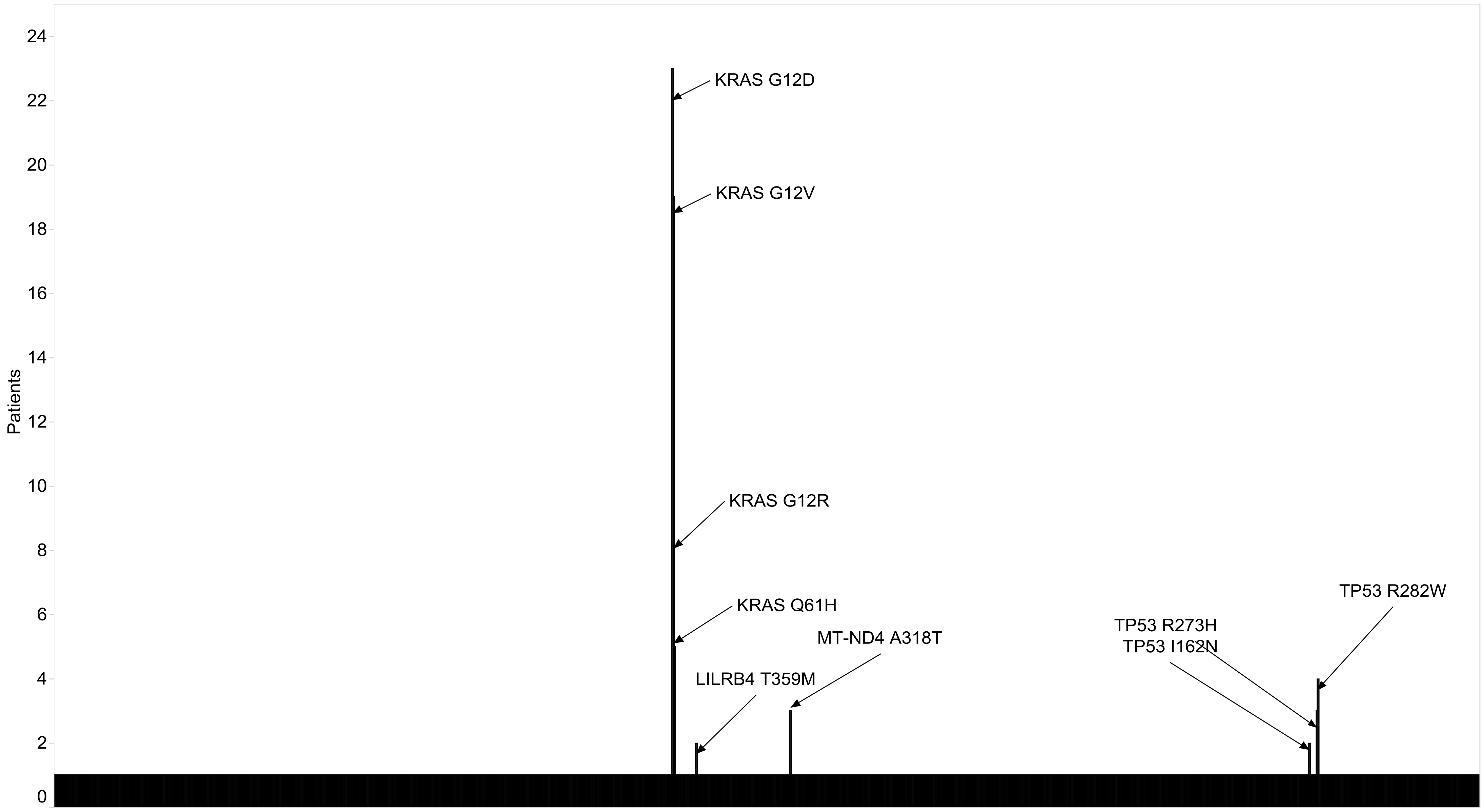

Supplementary Figure 2: Frequency of patients with predicted neoantigens. The x axis represents the mutations in ICGC PDAC samples for which we predicted all neopeptides. The y axis shows the number of patients who have < 500 nM binding affinity neopeptides from these mutations.

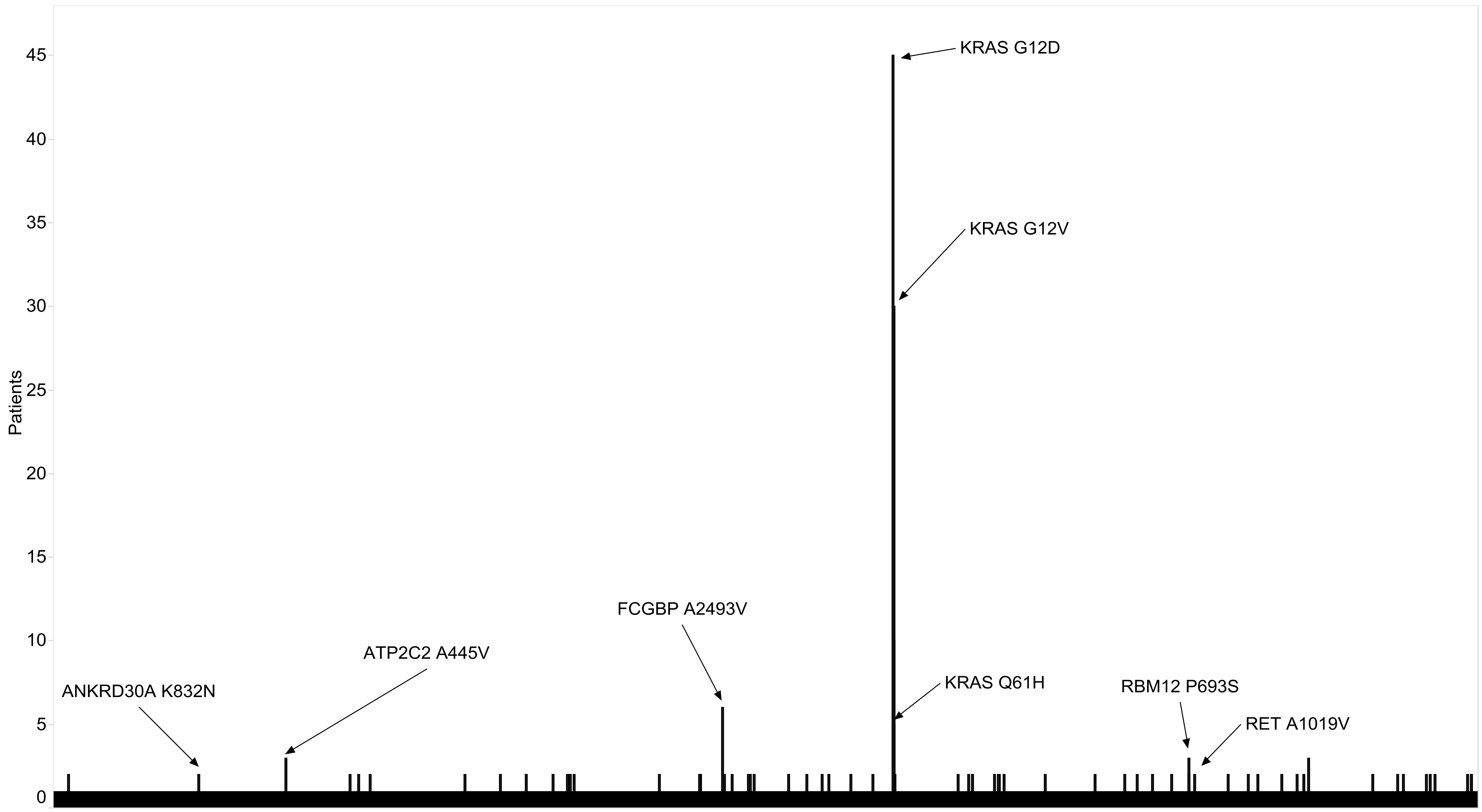

Supplementary Figure 2 (cont): Frequency of patients with predicted neoantigens. The x axis represents the mutations in TCGA PDAC samples for which we predicted all neopeptides. The y axis shows the number of patients who have < 500 nM binding affinity neopeptides from these mutations.

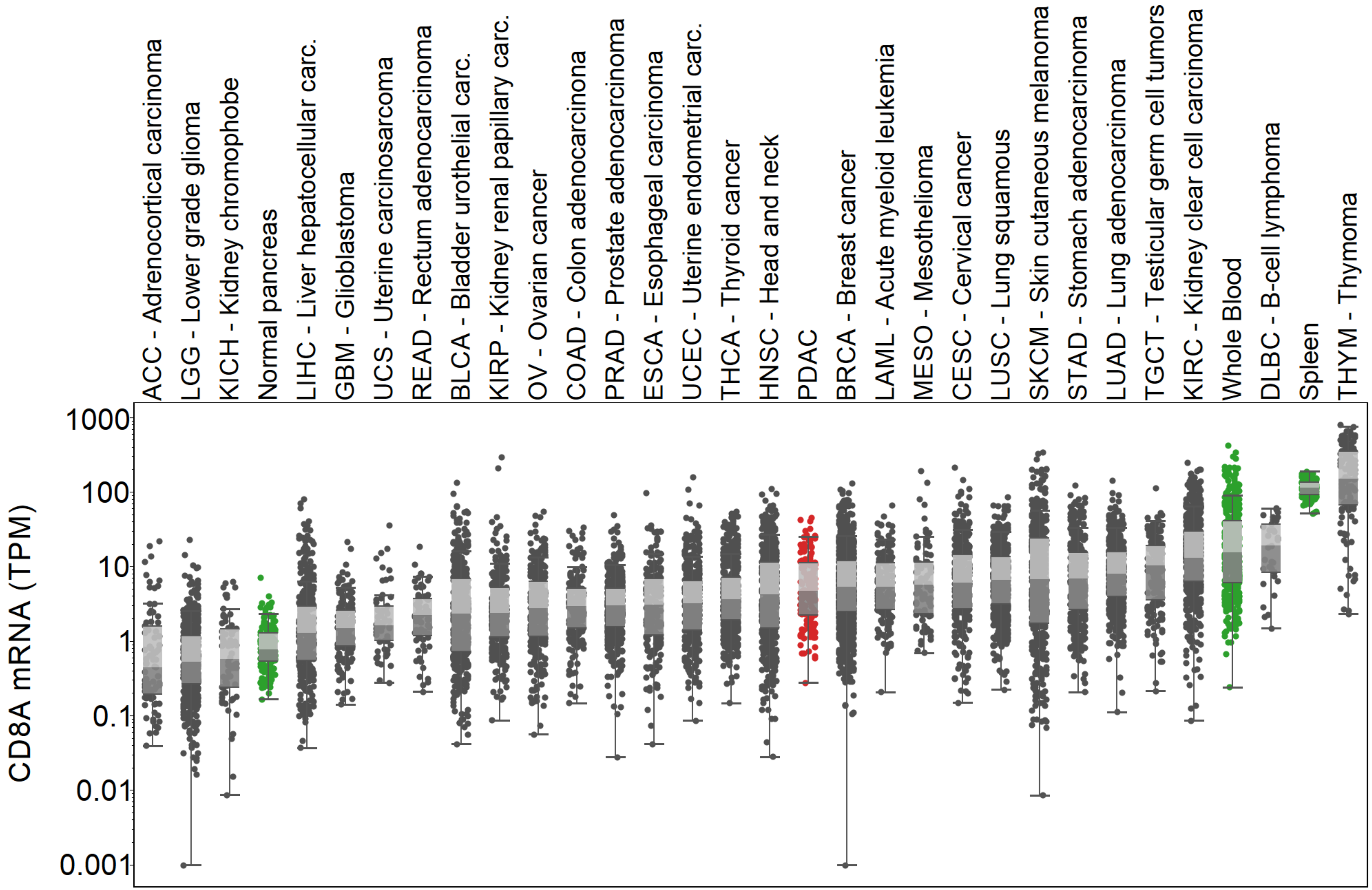

Supplementary Figure 3: Expression of **CD8A** in normal tissues (green) and tumors (grey) including pancreatic ductal adenocarcinoma (PDAC, red). Each dot represents a sample. Dark and light grey boxes represent the two quartiles around the median expression.

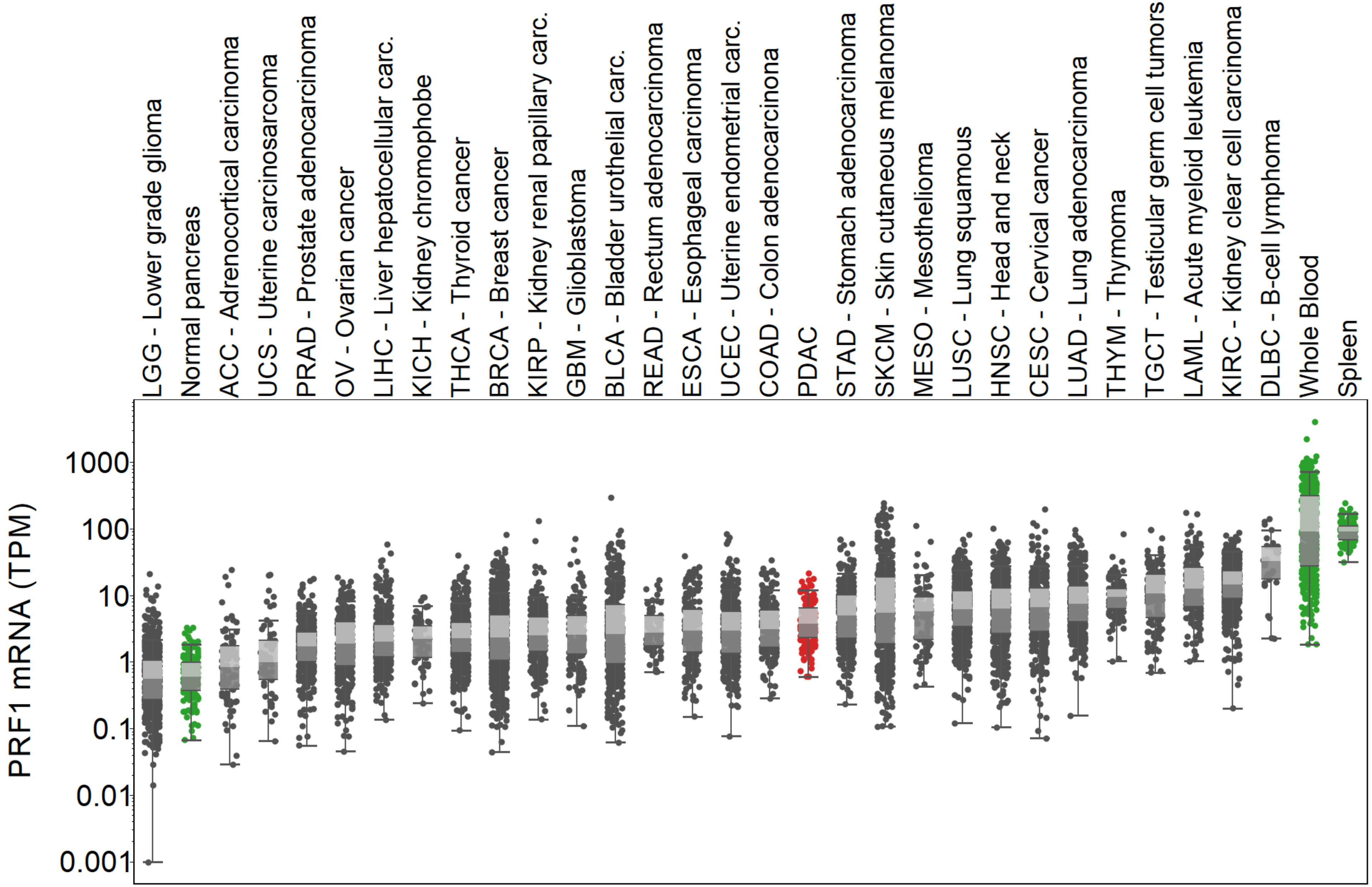

Supplementary Figure 3 (cont.): Expression of **PRF1** in normal tissues (green) and tumors (grey) including pancreatic ductal adenocarcinoma (PDAC, red). Each dot represents a sample. Dark and light grey boxes represent the two quartiles around the median expression.

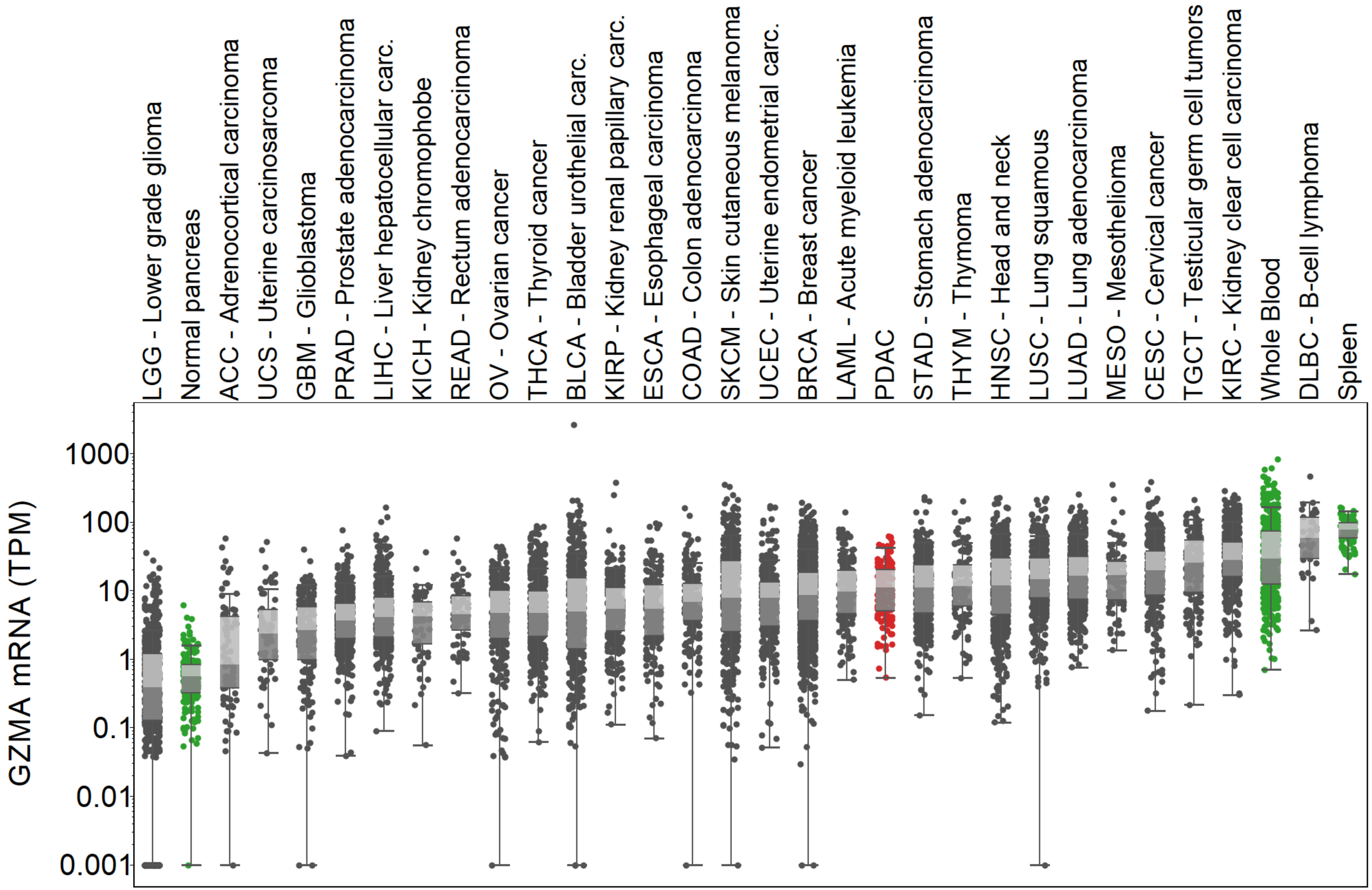

Supplementary Figure 3 (cont.): Expression of **GZMA** in normal tissues (green) and tumors (grey) including pancreatic ductal adenocarcinoma (PDAC, red). Each dot represents a sample. Dark and light grey boxes represent the two quartiles around the median expression.

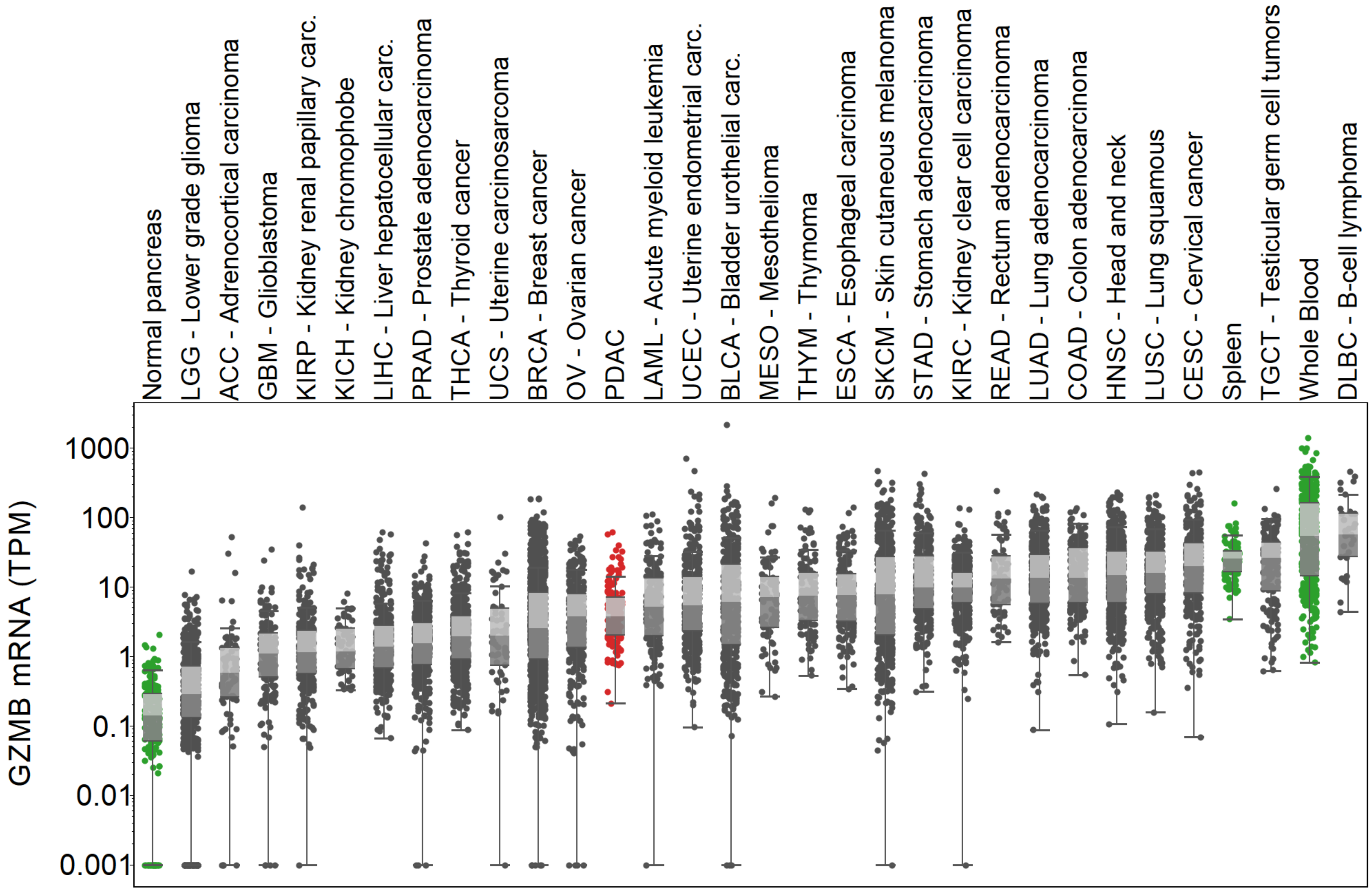

Supplementary Figure 3 (cont.): Expression of **GZMB** in normal tissues (green) and tumors (grey) including pancreatic ductal adenocarcinoma (PDAC, red). Each dot represents a sample. Dark and light grey boxes represent the two quartiles around the median expression.

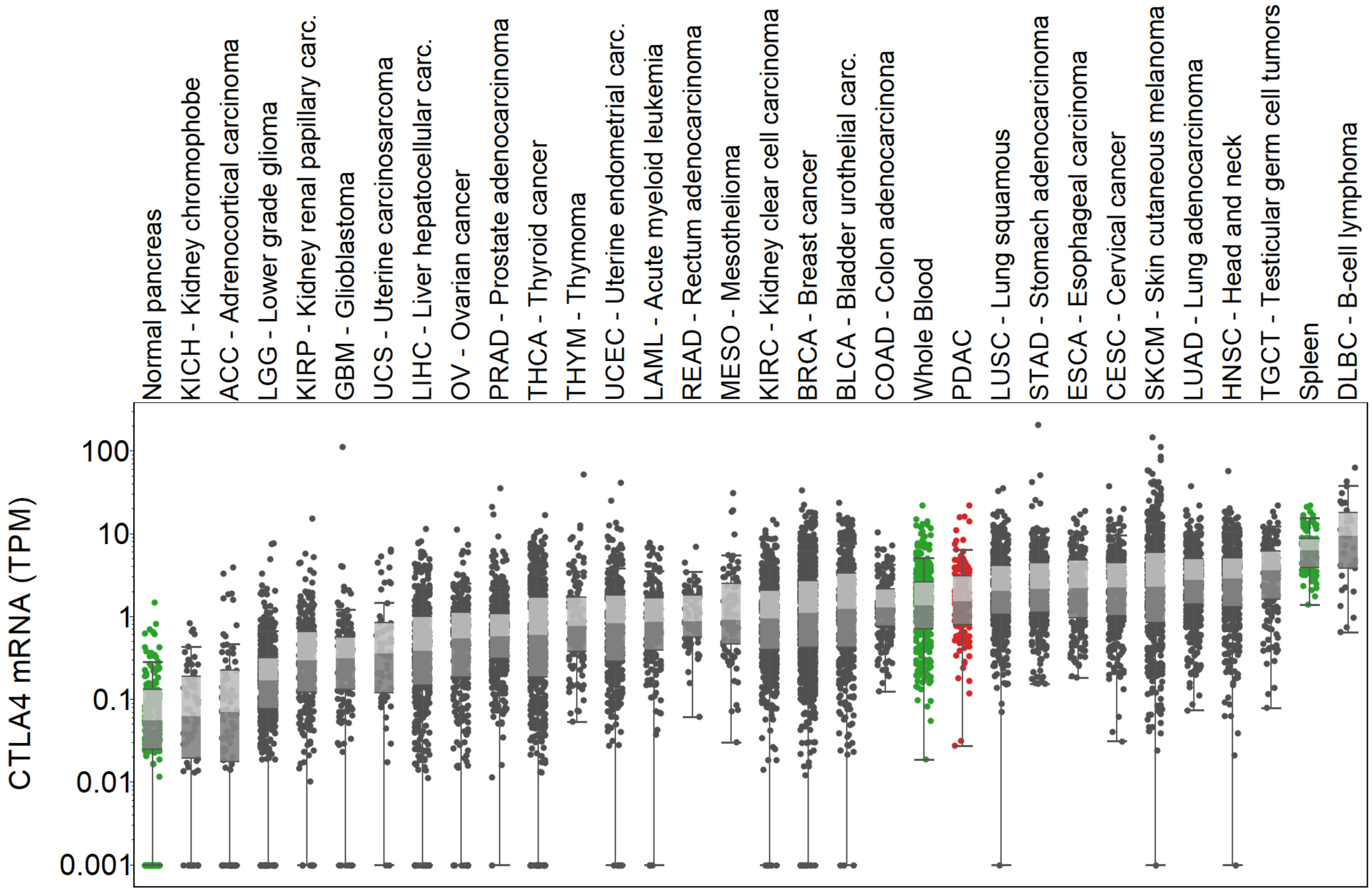

Supplementary Figure 3 (cont.): Expression of **CTLA4** in normal tissues (green) and tumors (grey) including pancreatic ductal adenocarcinoma (PDAC, red). Each dot represents a sample. Dark and light grey boxes represent the two quartiles around the median expression.

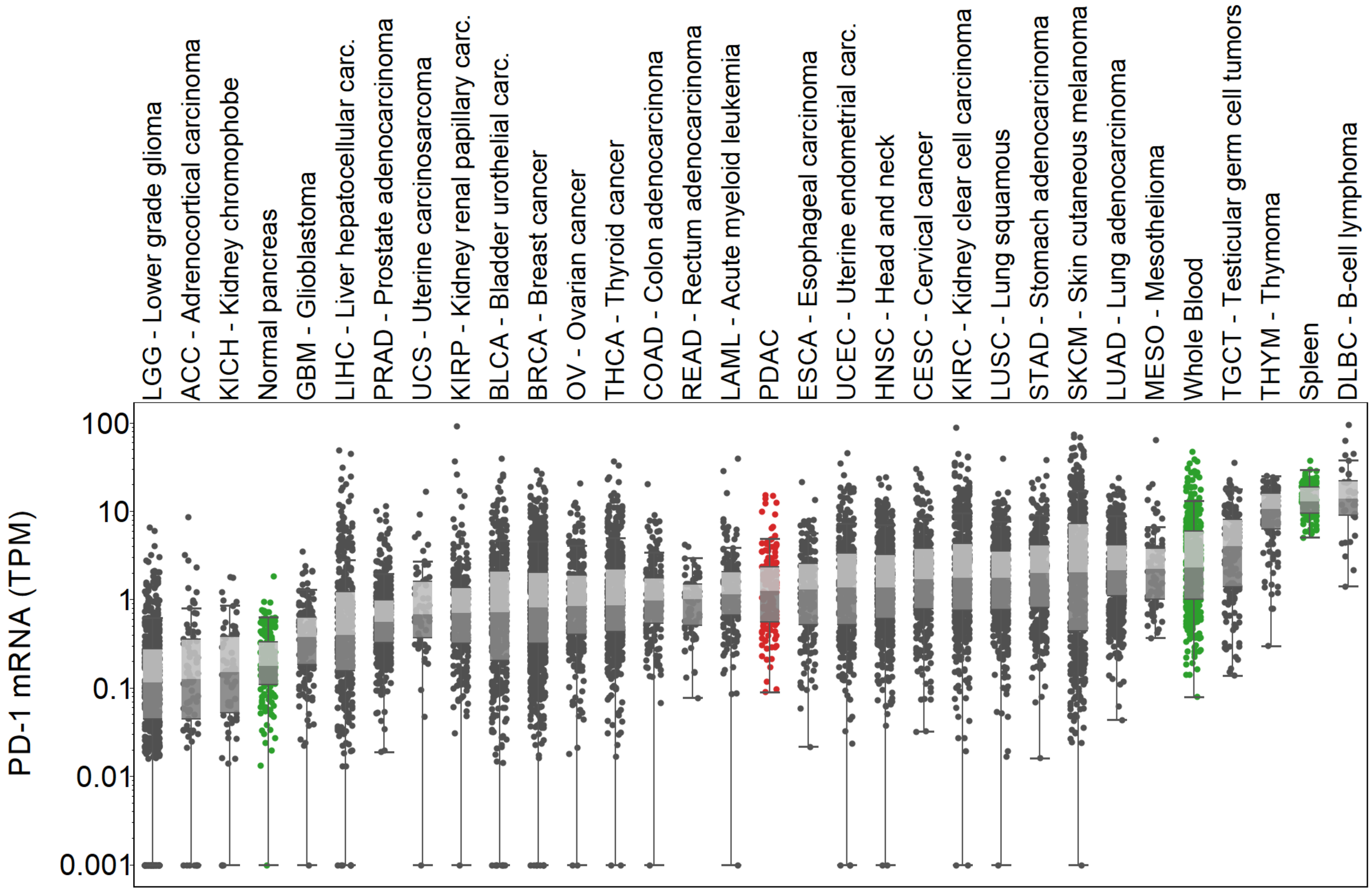

Supplementary Figure 3 (cont.): Expression of **PD-1** in normal tissues (green) and tumors (grey) including pancreatic ductal adenocarcinoma (PDAC, red). Each dot represents a sample. Dark and light grey boxes represent the two quartiles around the median expression.

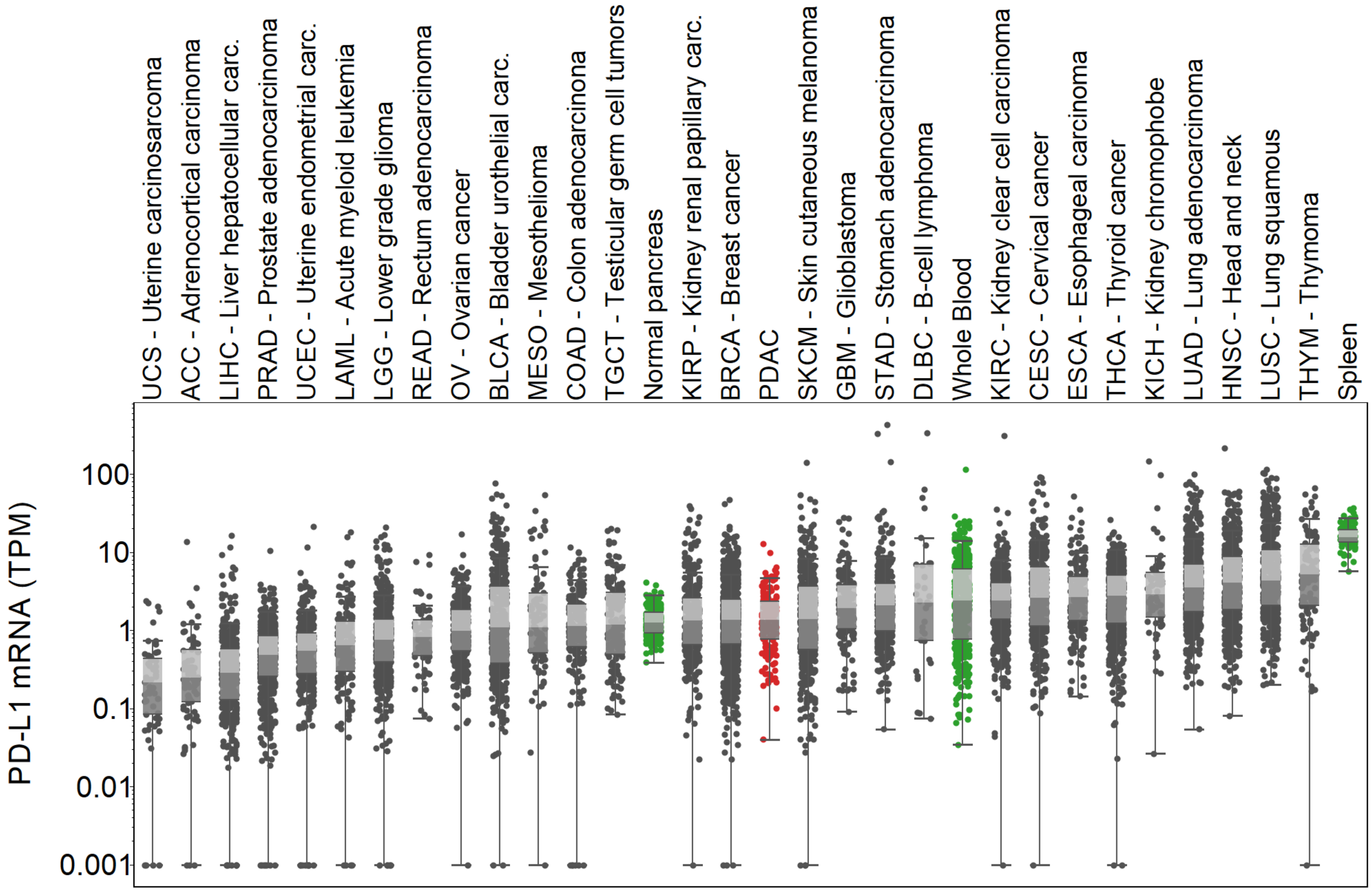

Supplementary Figure 3 (cont.): Expression of **PD-L1** in normal tissues (green) and tumors (grey) including pancreatic ductal adenocarcinoma (PDAC, red). Each dot represents a sample. Dark and light grey boxes represent the two quartiles around the median expression.

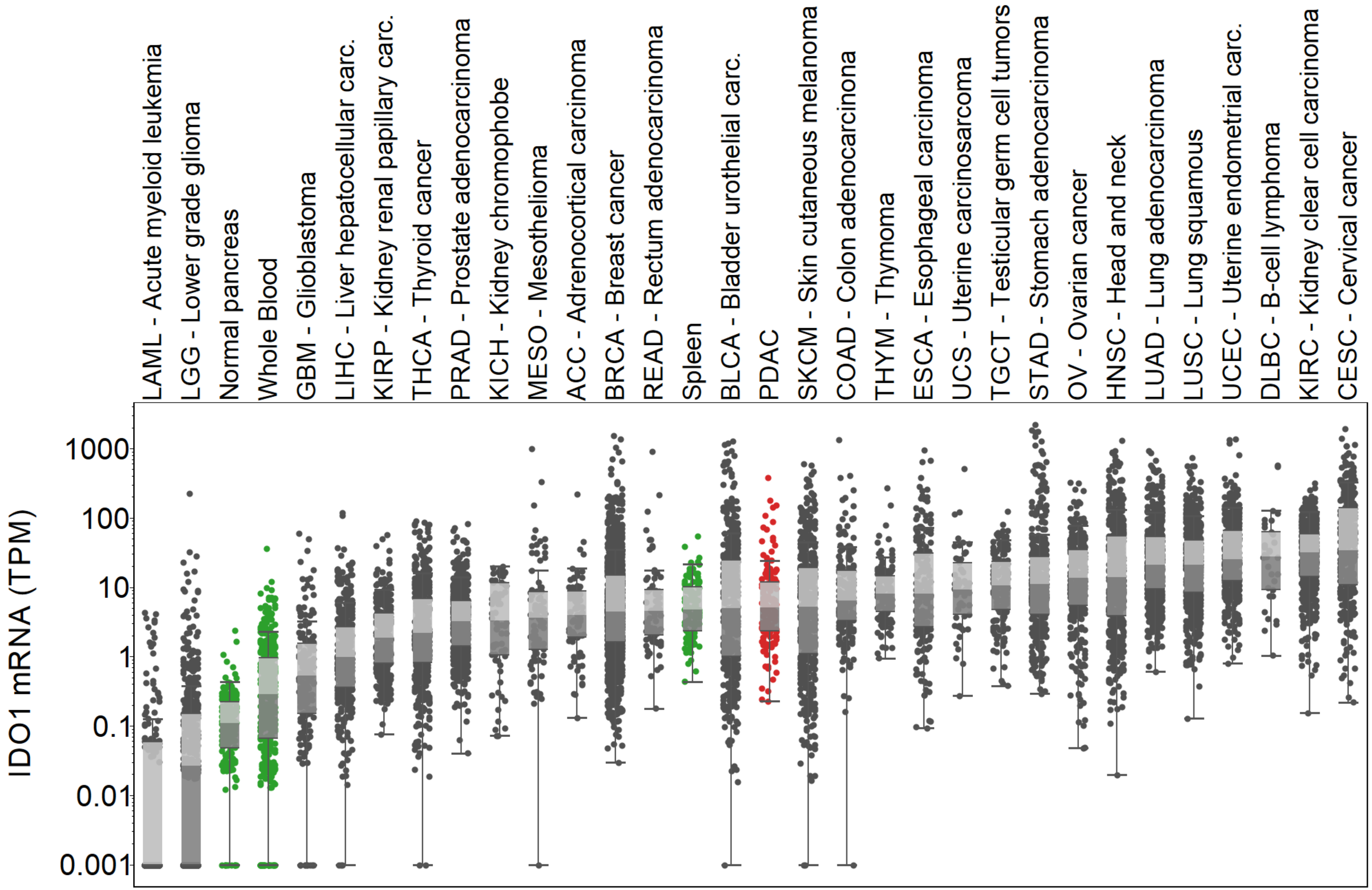

Supplementary Figure 3 (cont.): Expression of **IDO1** in normal tissues (green) and tumors (grey) including pancreatic ductal adenocarcinoma (PDAC, red). Each dot represents a sample. Dark and light grey boxes represent the two quartiles around the median expression.

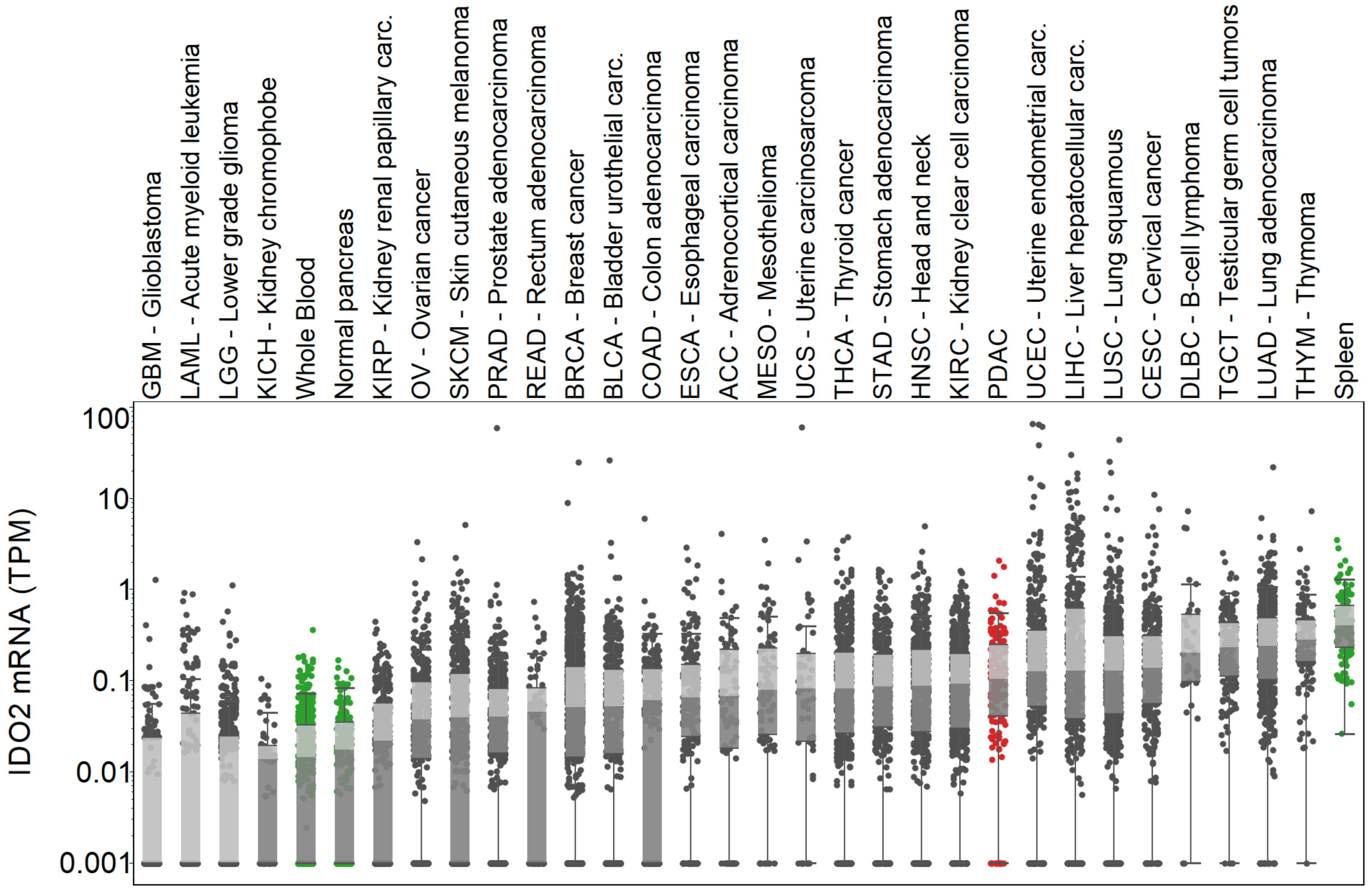

Supplementary Figure 3 (cont.): Expression of **IDO2** in normal tissues (green) and tumors (grey) including pancreatic ductal adenocarcinoma (PDAC, red). Each dot represents a sample. Dark and light grey boxes represent the two quartiles around the median expression.

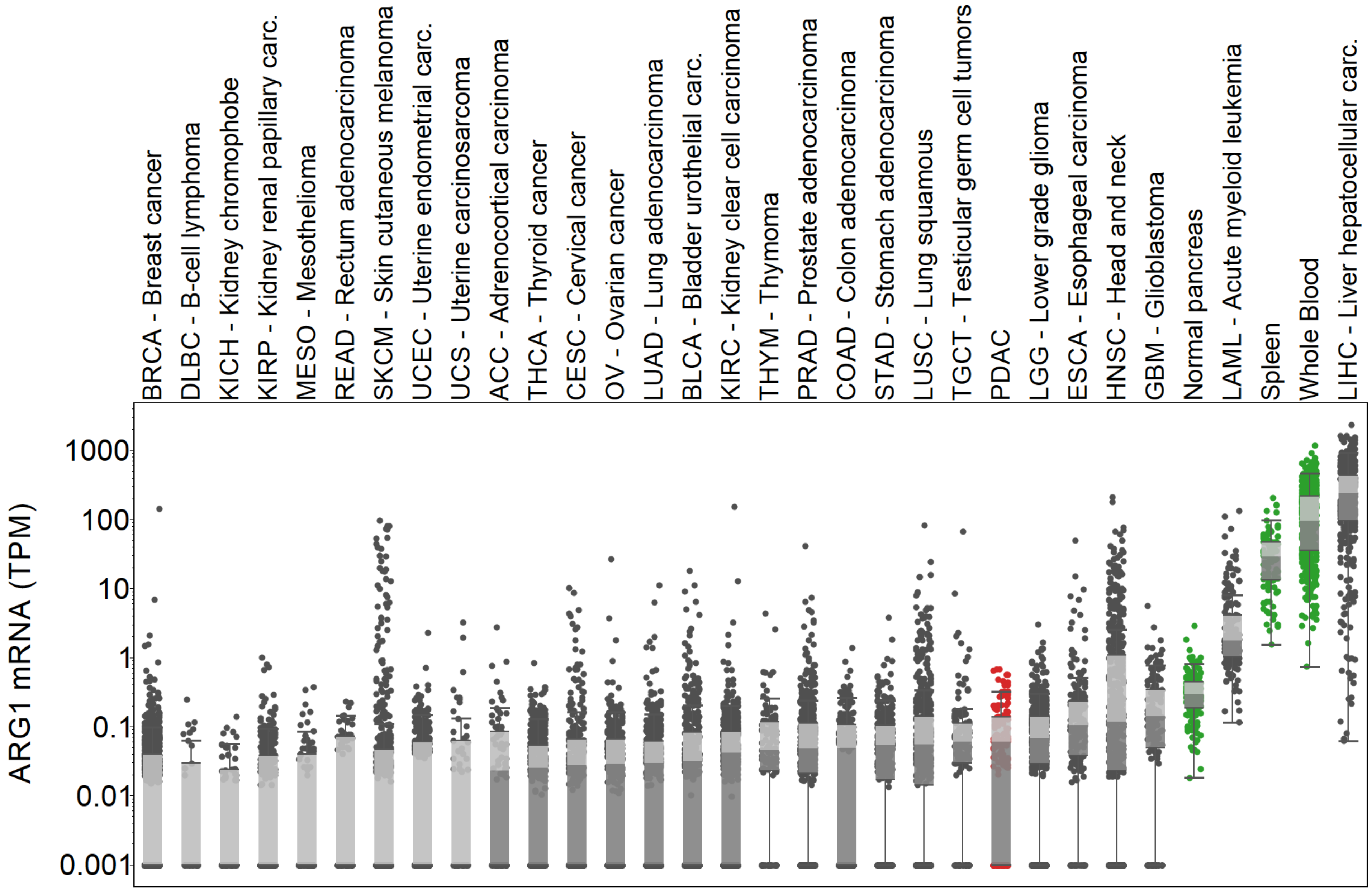

Supplementary Figure 3 (cont.): Expression of **ARG1** in normal tissues (green) and tumors (grey) including pancreatic ductal adenocarcinoma (PDAC, red). Each dot represents a sample. Dark and light grey boxes represent the two quartiles around the median expression.

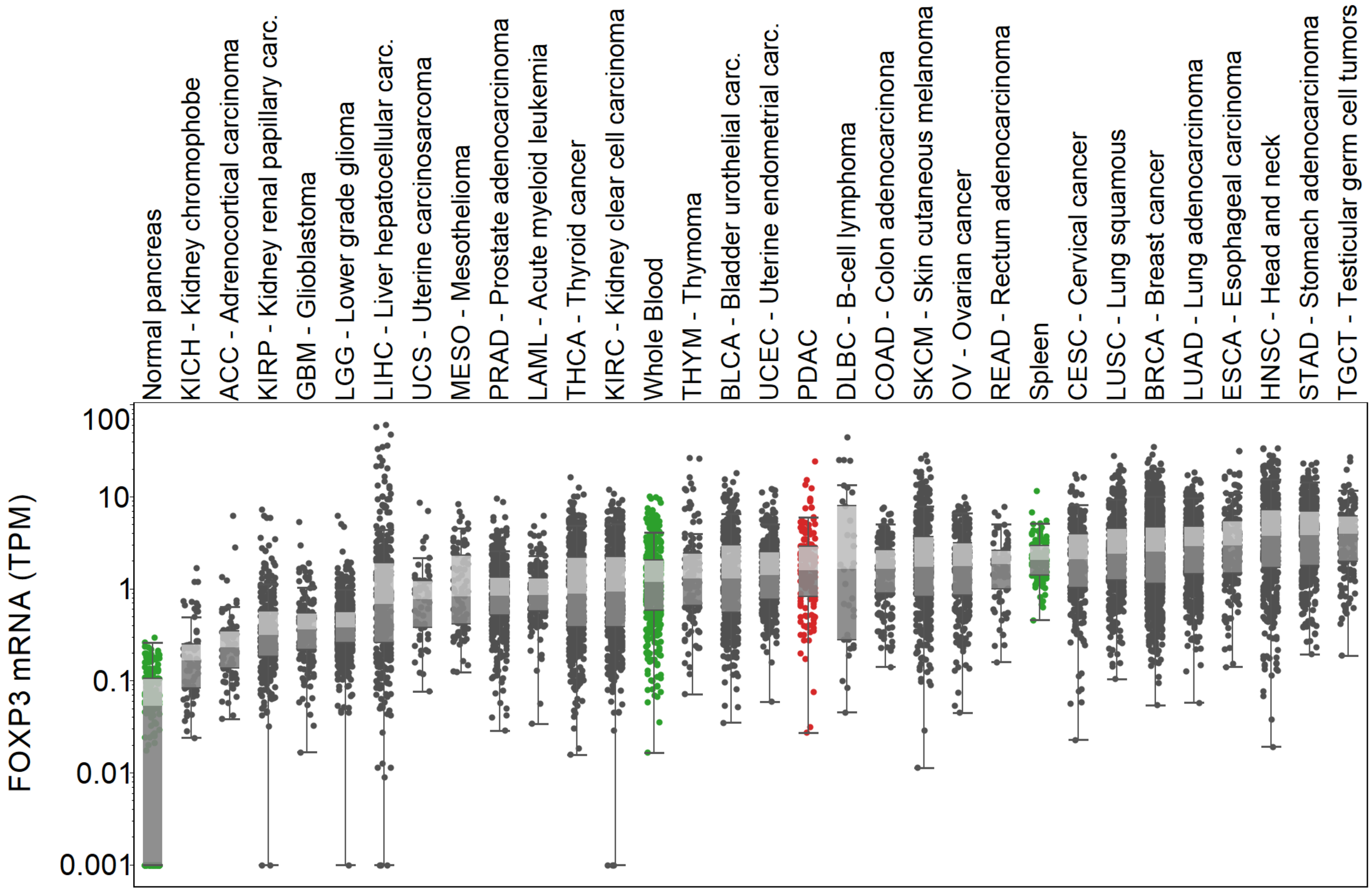

Supplementary Figure 3 (cont.): Expression of **FOXP3** in normal tissues (green) and tumors (grey) including pancreatic ductal adenocarcinoma (PDAC, red). Each dot represents a sample. Dark and light grey boxes represent the two quartiles around the median expression.

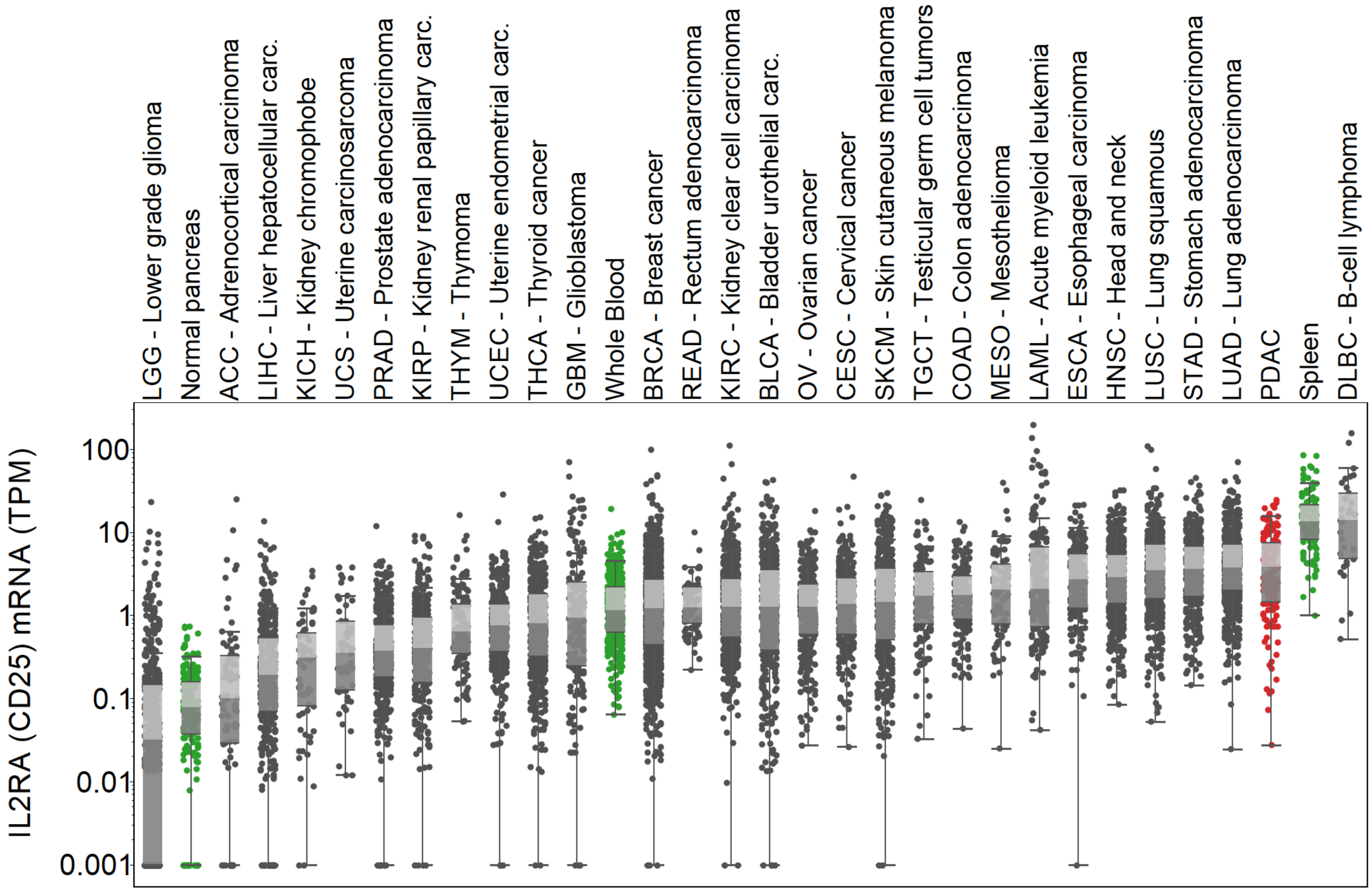

Supplementary Figure 3 (cont.): Expression of **IL2RA (CD25)** in normal tissues (green) and tumors (grey) including pancreatic ductal adenocarcinoma (PDAC, red). Each dot represents a sample. Dark and light grey boxes represent the two quartiles around the median expression.

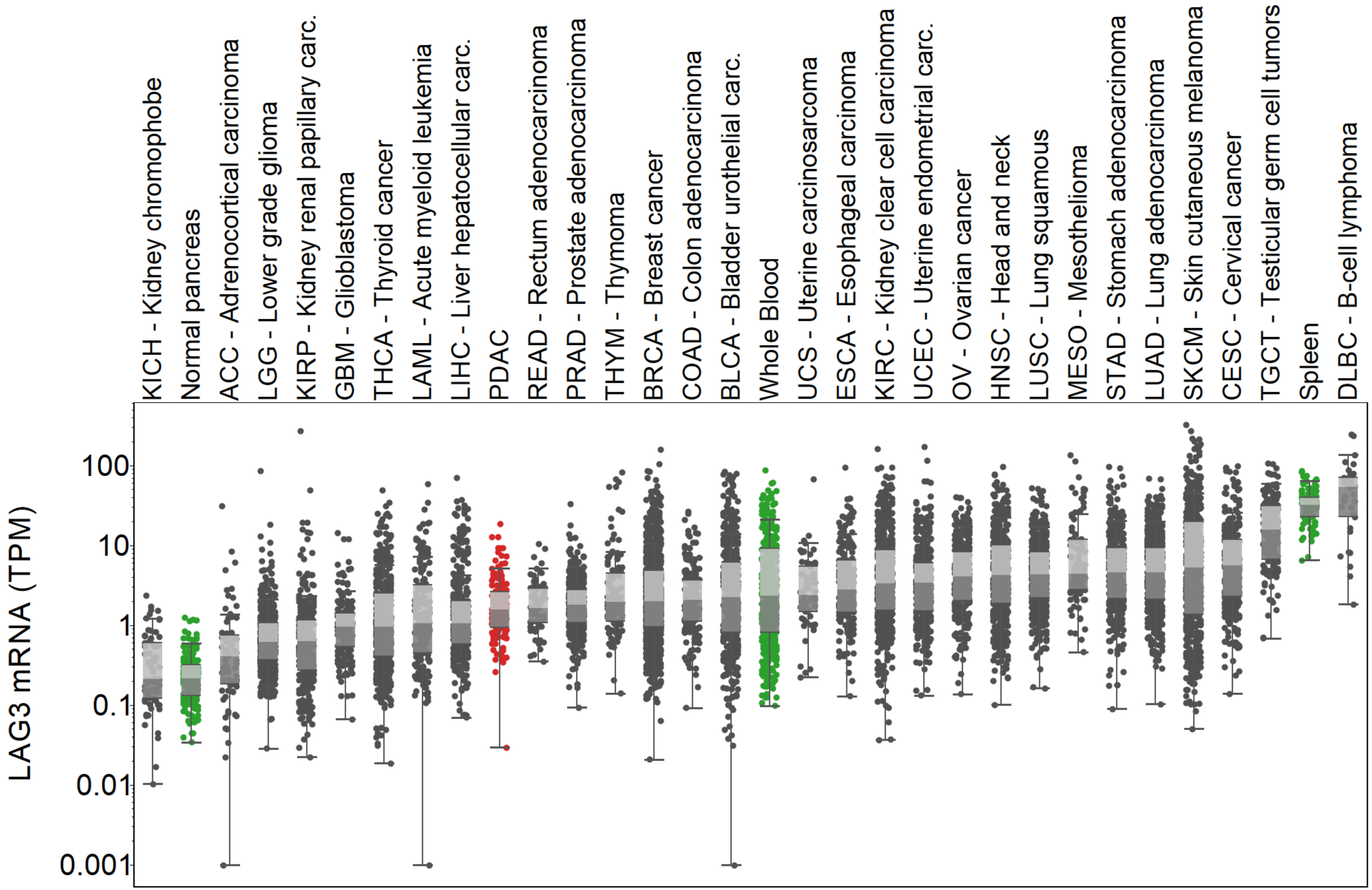

Supplementary Figure 3 (cont.): Expression of **LAG3** in normal tissues (green) and tumors (grey) including pancreatic ductal adenocarcinoma (PDAC, red). Each dot represents a sample. Dark and light grey boxes represent the two quartiles around the median expression.

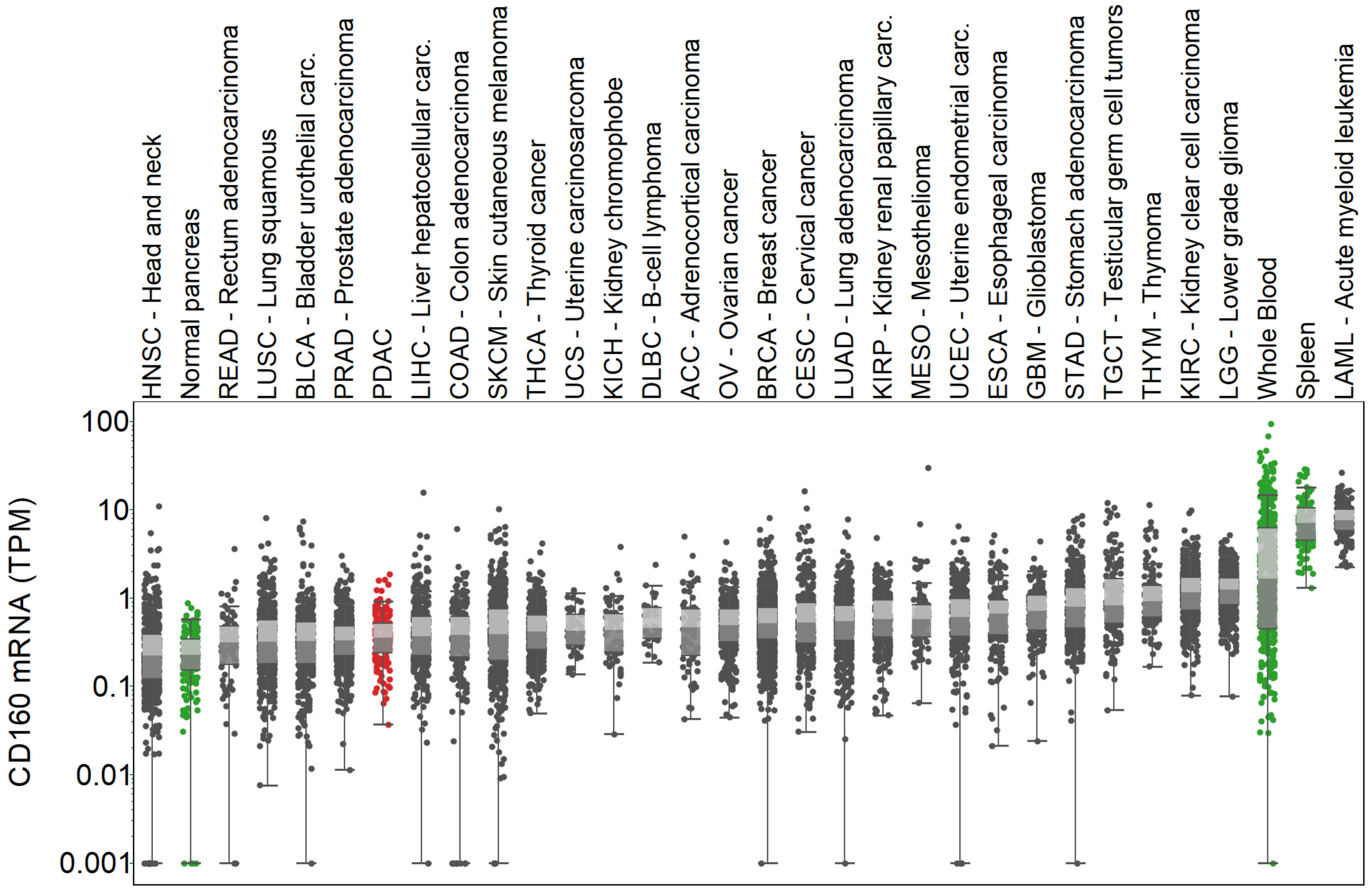

Supplementary Figure 3 (cont.): Expression of **CD160** in normal tissues (green) and tumors (grey) including pancreatic ductal adenocarcinoma (PDAC, red). Each dot represents a sample. Dark and light grey boxes represent the two quartiles around the median expression.

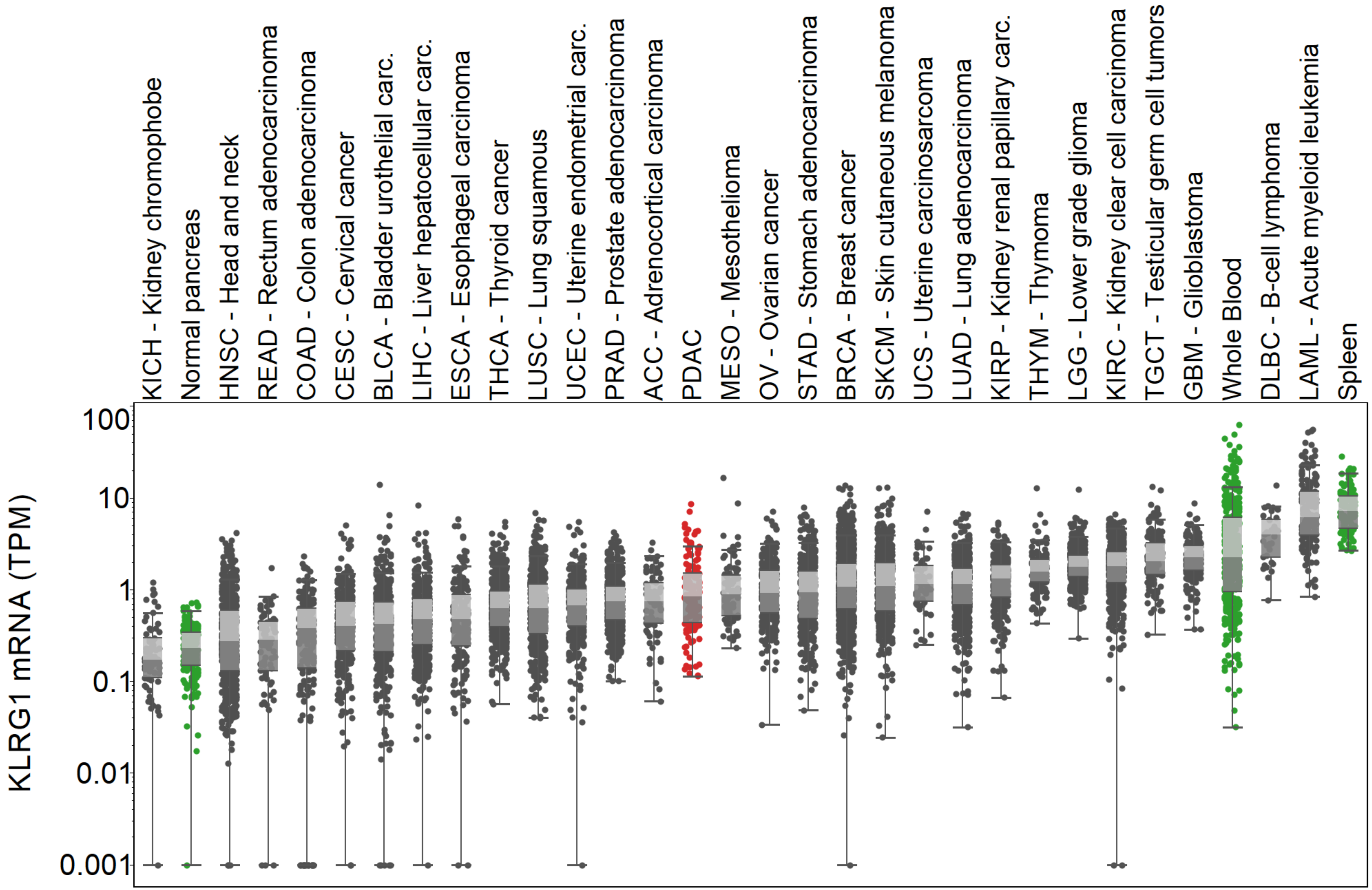

Supplementary Figure 3 (cont.): Expression of **KLRG1** in normal tissues (green) and tumors (grey) including pancreatic ductal adenocarcinoma (PDAC, red). Each dot represents a sample. Dark and light grey boxes represent the two quartiles around the median expression.

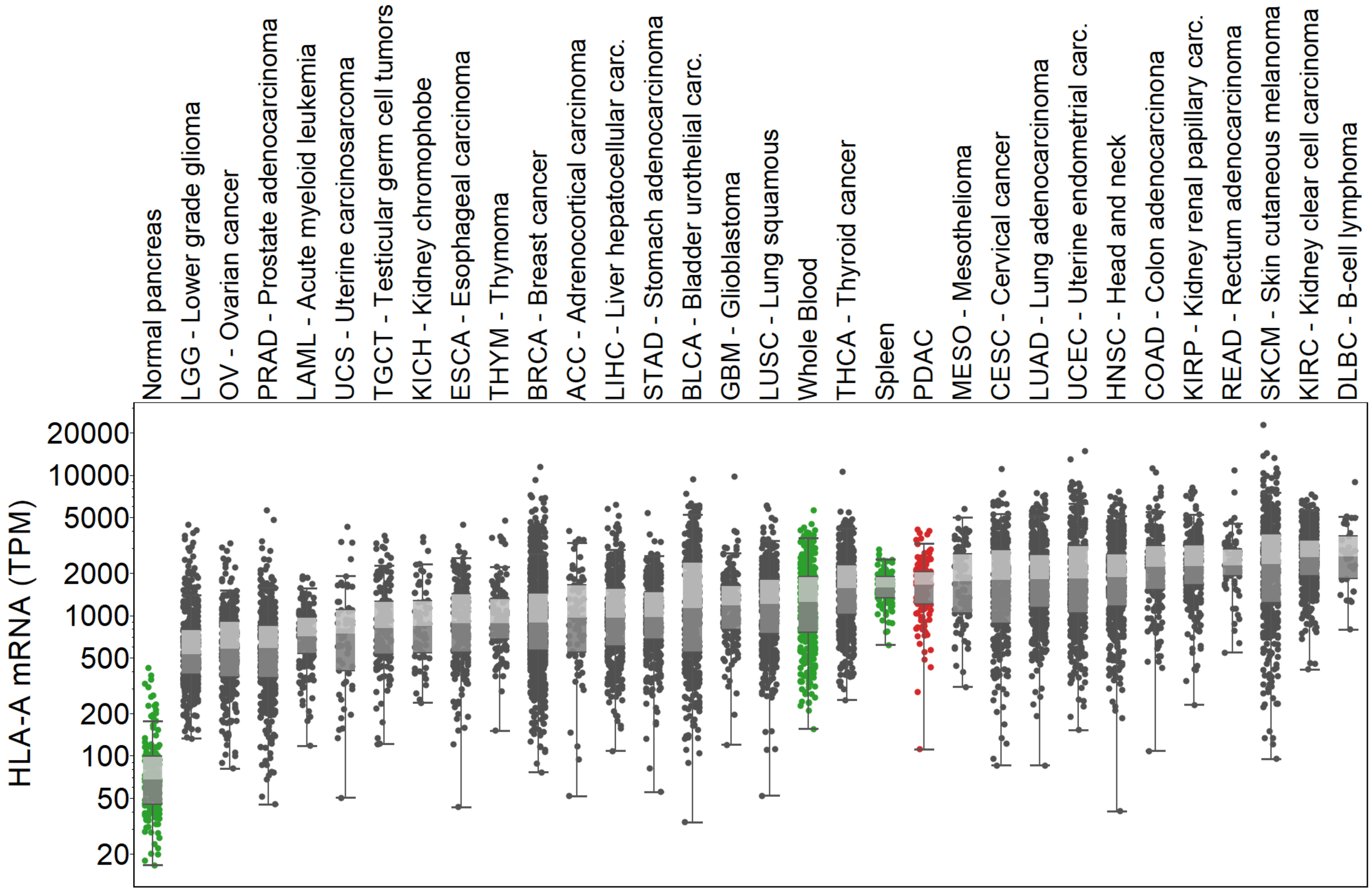

Supplementary Figure 3 (cont.): Expression of **HLA-A** in normal tissues (green) and tumors (grey) including pancreatic ductal adenocarcinoma (PDAC, red). Each dot represents a sample. Dark and light grey boxes represent the two quartiles around the median expression.

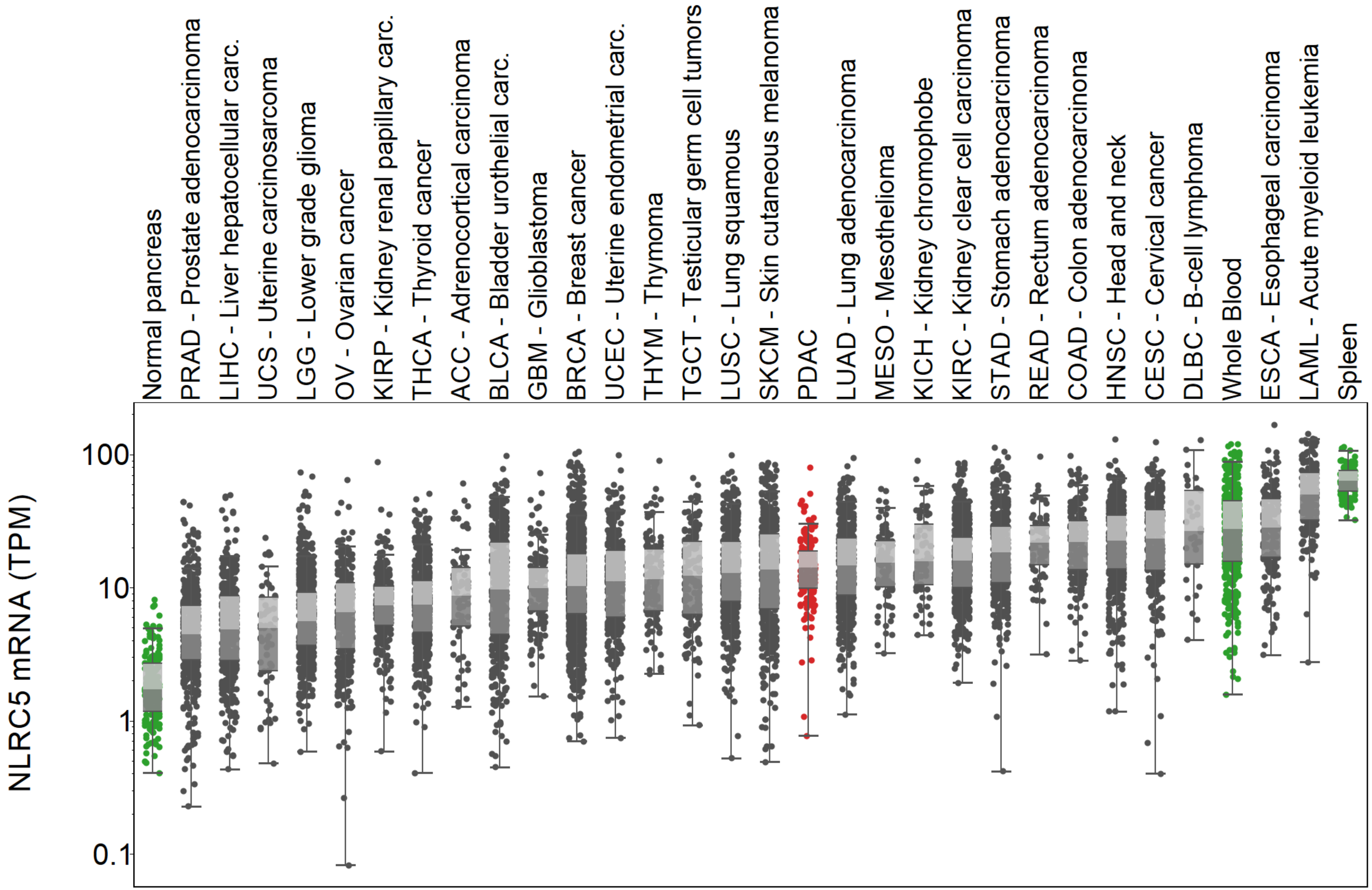

Supplementary Figure 3 (cont.): Expression of **NLRC5** in normal tissues (green) and tumors (grey) including pancreatic ductal adenocarcinoma (PDAC, red). Each dot represents a sample. Dark and light grey boxes represent the two quartiles around the median expression.

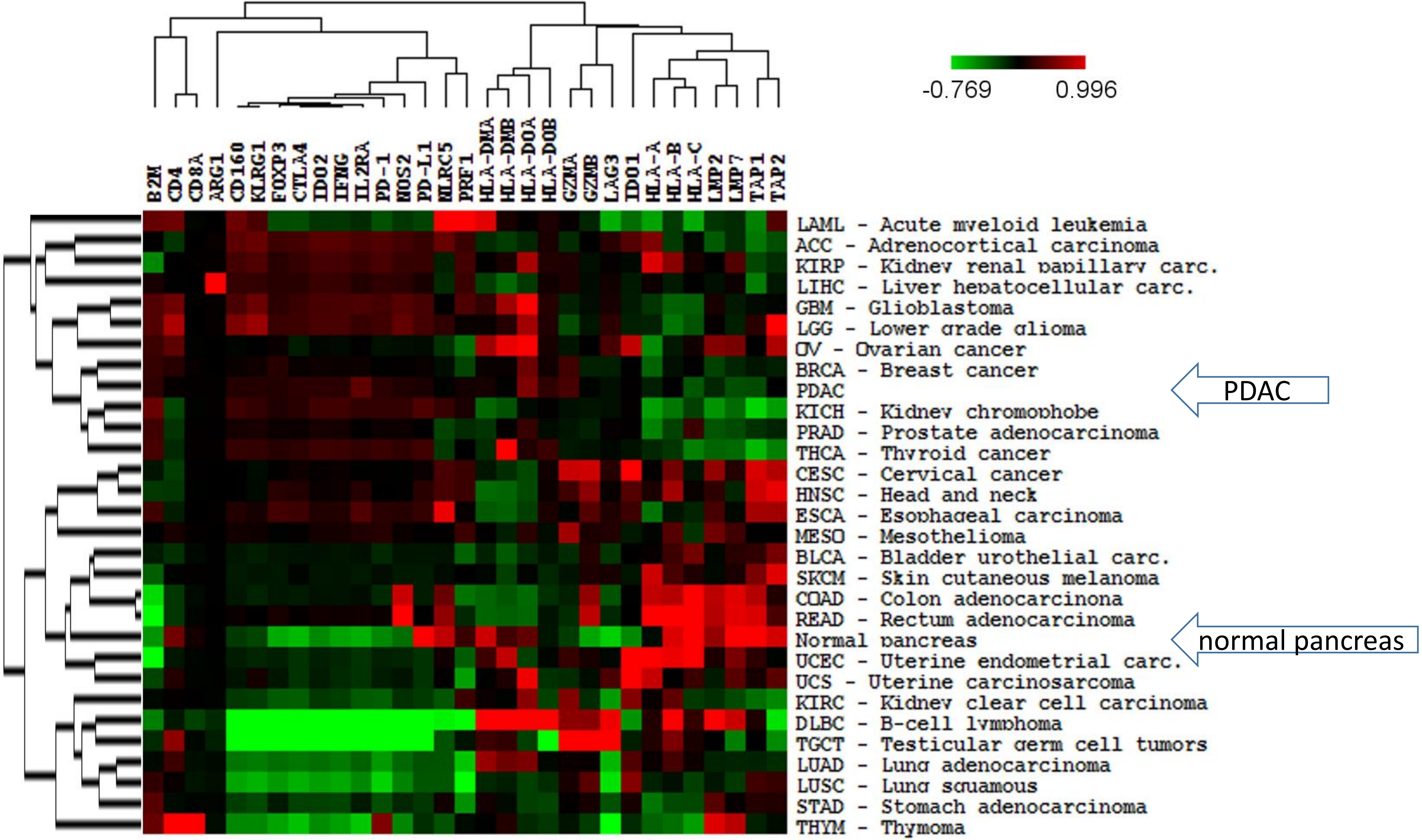

Supplementary Figure 3 (cont.): Clustering of median expression of genes related to antigen presentation, effector T cell function, and immune suppression in normal pancreas and tumors including pancreatic ductal adenocarcinoma (PDAC).

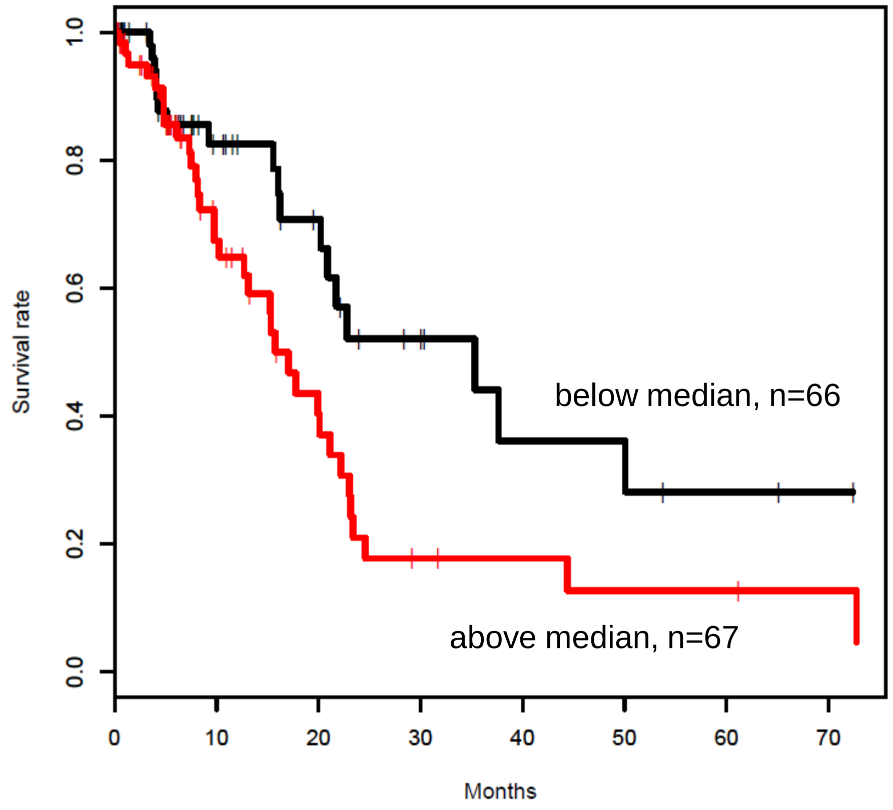

TCGA PDAC
($p < 0.05$)

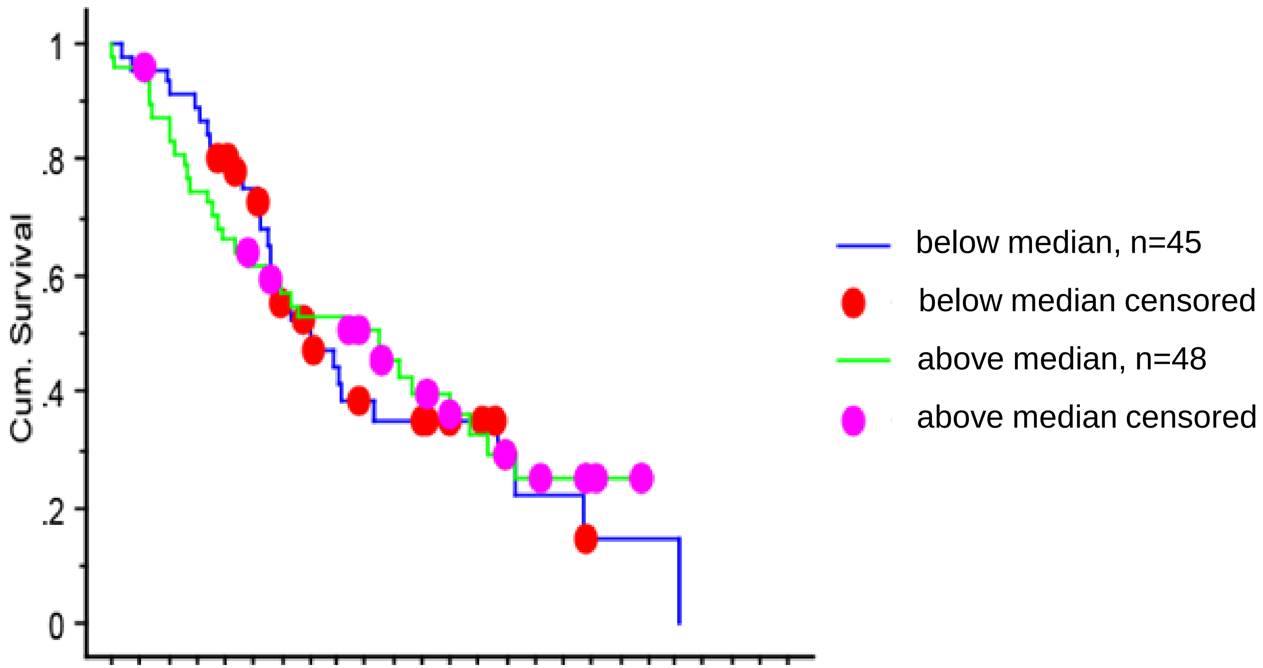

ICGC PDAC
(not significantly different)

Supplementary Figure 4: Kaplan-Meier analysis of overall survival and mutation load (below and above median mutation count) correlations.

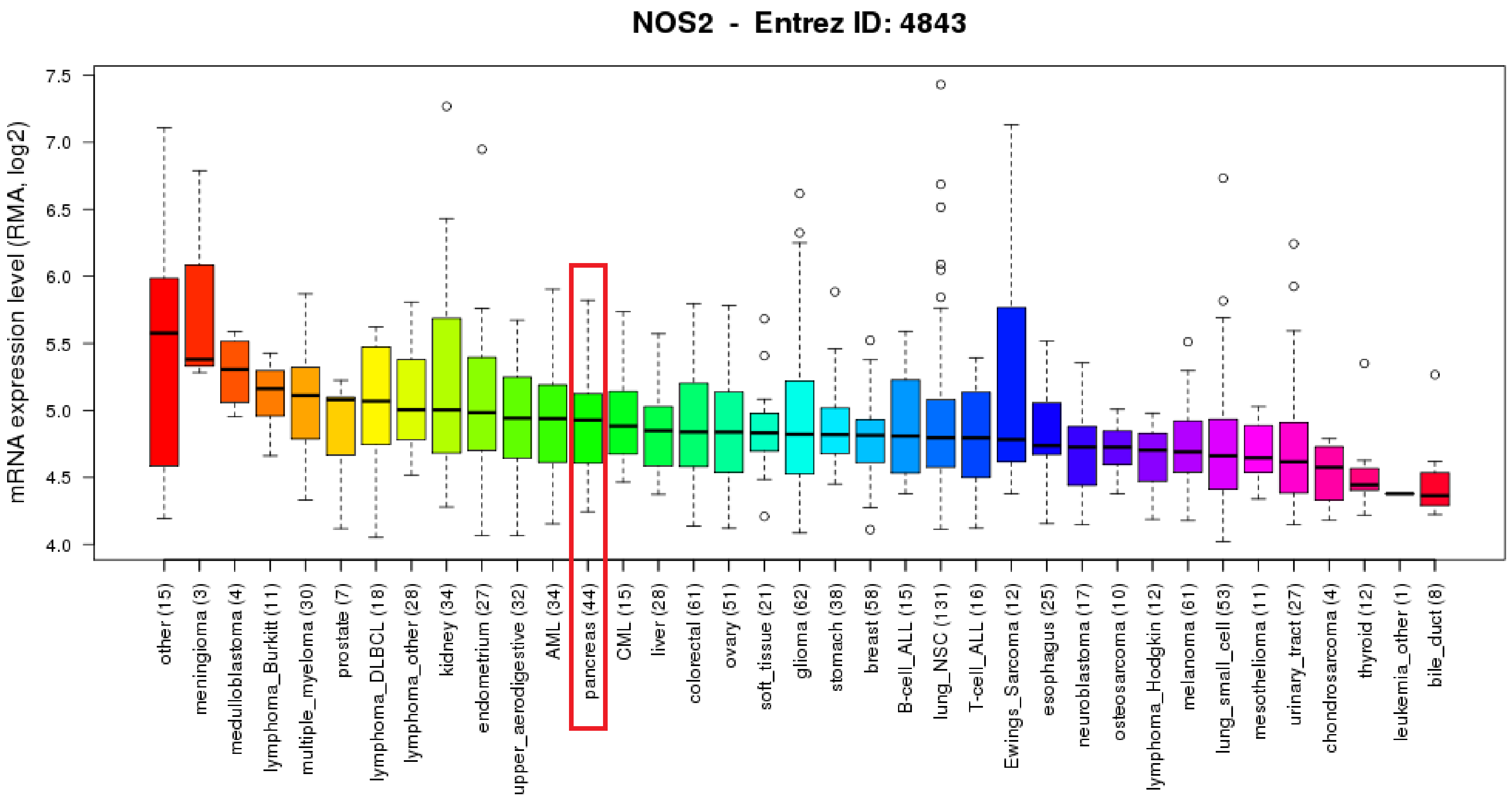

Supplementary Figure 5: Expression of NOS2 (iNOS) in the Cancer Cell Line Encyclopedia (modified from www.broadinstitute.org/ccle). Pancreatic cancer cell lines are shown in red rectangle.
